# Tailoring Alfalfa-Derived Biochar via Pyrolysis Temperature for Enhanced Adsorption of Diverse Per- and Polyfluoroalkyl Substances in Water

**DOI:** 10.1021/acsomega.6c06097

**Published:** 2026-10-22

**Authors:** Gi-Beom Kim, Soo Youn Lee, Sahng Hyuck Woo, Ying-Ren Lai, Eunsung Kan

**Affiliations:** † 14736 Texas A&M AgriLife Research and Extension Center, Stephenville, Texas 76401, United States; ‡ Yonsei University, Seodaemun-gu, Seoul 03722, Korea; § Gwangju Clean Energy Research Center, 140841 Korea Institute of Energy Research, Gwangju 61003, South Korea; ∥ Jeju Global Research Center, 65405 Korea Institute of Energy Research (KIER), 200 Haemajihaean-ro, Gujwa-eup, Jeju-si, Jeju-do 63359, Republic of Korea; ⊥ Department of Biological and Agricultural Engineering, Texas A&M University, College Station, Texas 77843, United States

## Abstract

This study investigated the removal of long- and short-chain per- and polyfluoroalkyl substances (PFAS) such as perfluorooctanoic acid (PFOA) and perfluorobutanoic acid (PFBA) using alfalfa-derived biochar produced at different pyrolysis temperatures (300–900 °C). Increasing pyrolysis temperature substantially altered the physicochemical properties of the biochars, resulting in higher surface area, higher fixed carbon content, lower O/C and H/C ratios, and more condensed carbon structures. Among the tested materials, the biochar produced at 900 °C (AF900) showed the highest adsorption performance for both PFOA (0.39417 mg/g BC) and PFBA (0.0972 mg/g BC). Adsorption was strongly pH-dependent, with the highest removal observed at pH 3.0, and PFOA consistently showed much greater adsorption than PFBA. Possible mechanisms may include electrostatic, hydrophobic, and π–π interactions. The batch adsorption studies indicated that PFOA was better fitted to the pseudo-second-order and Freundlich models, and PFBA to the Elovich and Temkin models, while the adsorption of PFOA and PFBA followed endothermic reactions. The reusability experiment for AF900 showed a gradual decrease in regeneration efficiency over three cycles for PFOA and PFBA, with a relatively higher decrease for PFBA. Adsorption of PFOA and PFBA on AF900 in groundwater was lower than that in DI water, although increasing the loading rate of AF900 substantially improved PFAS removal in groundwater. Overall, AF900 was the best-performing material for adsorption of PFOA and PFBA among the alfalfa-derived biochars prepared at various temperatures in this study, whereas its removal performance in groundwater needs to be further studied.

## Graphical abstract

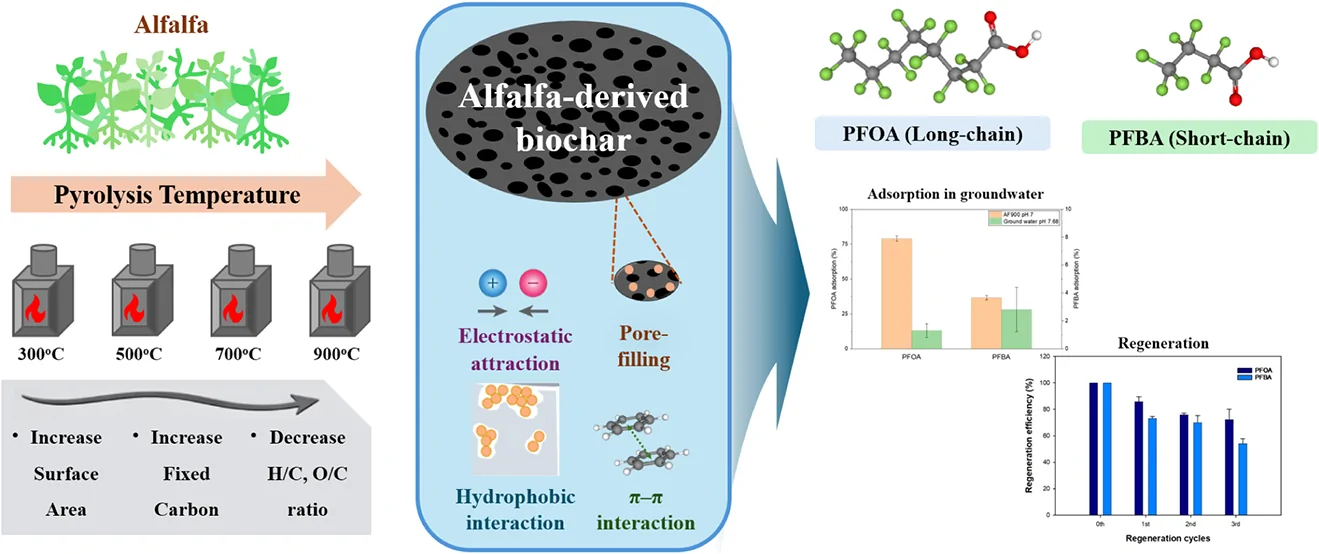

## 1 Introduction

Per- and polyfluoroalkyl substances (PFAS) are a major class of persistent organic pollutants of global concern because of their extremely strong C–F bonds, exceptional chemical stability, and resistance to degradation in natural environments.^1^ Due to their high persistence, PFAS continuously accumulate in water bodies, soils, sediments, wildlife, and even human serum.^2^ Over several decades, extensive industrial use in various products, including firefighting foams, textiles, coatings, and consumer goods, has resulted in their widespread contamination in surface water, groundwater, drinking water, and food.^3^ Long-term exposure to PFAS has been associated with carcinogenic effects, endocrine disruption, immunotoxicity, metabolic disorders, and developmental toxicity.^4^ These risks highlight an urgent need to develop treatment technologies that can effectively remove PFAS even at trace concentrations.

Various technologies have been investigated for PFAS remediation, including biodegradation, advanced oxidation and reduction, electrochemical degradation, photocatalysis, membrane filtration, and thermal treatment. However, their practical application can be limited by slow treatment rates, high energy requirements, byproduct formation, and reduced efficiency in complex wastewater matrices.^5^ Adsorption has therefore been widely used as a relatively simple and cost-effective treatment option with potential for large-scale application.^6,7^ Activated carbon (AC) is one of the most used adsorbents for PFAS removal. Its adsorption is strongly influenced by hydrophobic interactions, resulting in better removal of long-chain PFAS such as perfluorooctanoic acid (PFOA) than of less hydrophobic short-chain PFAS such as perfluorobutanoic acid (PFBA).^8,9^ This limitation highlights the need to investigate alternative sorbents that can provide nonhydrophobic interaction pathways, such as electrostatic attraction, hydrogen bonding, π–π interaction, and pore filling. However, whether these mechanisms are sufficient to compensate for the intrinsically weak hydrophobic affinity of short-chain PFAS remains uncertain.

Biochar (BC) is a carbonaceous material produced by pyrolysis of biomass and other organic feedstocks. Its relatively low production cost and variable physicochemical properties have led to increasing interest in its use as an adsorbent.^10,11^ Depending on the feedstock and pyrolysis temperature, BC may contain inorganic minerals, metal oxides, and surface functional groups such as hydroxyl (−OH), carboxyl (−COOH), and carbonyl (C=O) groups.^12^ These features can facilitate electrostatic attraction, coordination, or other specific interactions with long and short-chain PFAS.^13,14^

However, to the best of our knowledge, the application of alfalfa-derived biochar (AF-BC) for the adsorption of PFAS from aqueous solutions has received relatively limited attention, although AF-BC has been investigated for the adsorption of other emerging contaminants in water.^15–17^

As one of the most widely cultivated forage crops globally, alfalfa provides an abundant and stable supply of agricultural residues.^18^ Its balanced lignocellulosic composition and its high contents of inorganic minerals promote the formation of carbon and various metal oxides in biochar during pyrolysis.^19,20^ These unique properties can provide broad and effective functional groups that differ from other types of biochars, and may be more favorable for the removal of PFAS.^21,22^ Despite these promising attributes of AF-BC for PFAS, the relationship between pyrolysis temperature, biochar properties, and PFAS adsorption performance of AF-BCs has not been comprehensively explored.^17,23,24^

Furthermore, this study provides detailed and comparative insights into the adsorption behaviors of long- and short-chain PFAS in relation to changes in AF-BC properties associated with different pyrolysis temperatures. Although several studies investigated the effects of pyrolysis temperature on biochar properties and adsorption of PFAS,^25,26^ relatively limited studies have been made to systematically examining the relationships among pyrolysis temperature, resulting biochar properties, and the adsorption behaviors of both long- and short-chain PFAS. Therefore, a more comprehensive understanding of how pyrolysis temperature-induced changes in biochar properties influence the adsorption of PFAS with different chain lengths is still needed.

This study produced nonactivated AF-BCs at 300, 500, 700, and 900 °C and characterized their physicochemical properties. Their adsorption performance for PFOA and PFBA was then evaluated through batch experiments, together with the possible adsorption mechanisms and practical applicability. PFOA and PFBA were selected as representative long- and short-chain perfluoroalkyl carboxylic acids, respectively. These compounds possess the same terminal carboxylate functional group but differ substantially in their overall carbon-chain lengths (C8 for PFOA and C4 for PFBA), allowing the influence of chain length and hydrophobicity on adsorption behavior to be examined. The findings from this study provide a comprehensive evaluation of the relationships between pyrolysis temperature, physicochemical properties, and the adsorption behavior of nonactivated AF-BCs toward both long-chain and short-chain PFAS, contributing to the development of sustainable and low-cost sorbents for PFAS remediation.

## 2 Materials and Methods

### 2.1 Feedstock and Chemicals

Alfalfa (*Medicago sativa* *L*.) used in this study was obtained from a local feed supplier (The Hay and Feed Ranch, Stephenville, TX, USA). PFOA and PFBA were purchased from Fisher Scientific (Waltham, MA). Methanol and acetonitrile were obtained from Fisher Scientific.

### 2.2 Preparation of Alfalfa-Derived Biochar

Alfalfa (AF), including both stems and leaves, was oven-dried at 60 °C overnight and subsequently ground, sieved to a particle size below 500 μm prior to pyrolysis. Approximately 15 g of the ground AF was placed into a pyrolysis reactor (Multi Position Tube Furnace GSL-1100X-S, MTI Co., Richmond, Ca, USA). Pyrolysis was carried out under a continuous nitrogen atmosphere at target temperatures of 300, 500, 700, and 900 °C. The temperature was increased at a heating rate of 10 °C min^–1^ and maintained at the target temperature for 2 h. After pyrolysis, BC was suspended in DI water and stirred for 24 h to remove water-soluble impurities. Subsequently, the sample was treated with 0.1 M HCl solution (1.0 g BC per 40.0 mL HCl) for 30 min. This acid treatment was conducted to eliminate residual impurities, including inorganic salts and ash components, from the biochar.^27,28^ Then, DI water was used to elute the remaining HCl. The biochar suspension under stirring was adjusted by 1.0 M NaOH to pH 7.0. The solid residue was recovered by filtration and subsequently dried in an oven at 60 °C. The produced biochars were labeled according to the feedstock and the corresponding pyrolysis temperature (e.g., AF300, AF500, AF700, and AF900). The resulting biochars were then ground and sieved to the size below 106 μm for further experiments.

### 2.3 Characterization of Alfalfa-Derived Biochar

The specific surface area and pore size distribution of the biochars produced at different pyrolysis temperatures (AF300, AF500, AF700, and AF900) were determined using a BET surface area analyzer at Rice University (TX, USA). The elemental composition of the biochars, including carbon (C), hydrogen (H), and nitrogen (N), was analyzed using an elemental analyzer (Robert Microlit Lab, NJ). Proximate analysis was performed to determine the contents of fixed carbon, volatile matter, and ash following the ASTM D7582-12 standard method.^29^ The surface functional groups of the biochars were examined using Fourier transform infrared spectroscopy (FT-IR; IRPrestige-21, Shimadzu, Japan) and X-ray photoelectron spectroscopy (XPS; EnvironESCA spectrometer, Berlin, Germany), while the crystalline structure was investigated by X-ray diffraction (XRD; MiniFlex II, Rigaku Corp., Tokyo, Japan).

The point of zero charge (pHpzc) of AF900 was determined using the pH drift method.^30^ In brief, 50 mL of 0.01 M NaCl solution was placed in a 100 mL serum bottle, and the initial pH was adjusted between 3 and 10 using 0.1 M NaOH or HCl solutions. Afterward, 0.01 g of AF900 was introduced into the NaCl solution, and the headspace of the bottle was purged with nitrogen gas to remove dissolved CO_2_. The suspension was then equilibrated at 22 °C for 48 h, after which the final pH was recorded. The pH_pzc_ was identified as the pH at which the difference between the final and initial pH values (ΔpH = final pH – initial pH) was equal to zero.

### 2.4 Batch Adsorption of PFAS

Batch adsorption experiments were conducted to investigate the effects of solution pH on the adsorption of PFOA and PFBA using the biochar produced at different peak pyrolysis temperatures (AF300, AF500, AF700, and AF900). Briefly, 40 mL of PFOA and PFBA solutions (100 μg/L) prepared in deionized (DI) water were added to 50 mL polypropylene centrifuge tubes, followed by the addition of 10 mg of biochar. The suspensions were adjusted to pH values ranging from 3 to 9 and agitated at 23 °C for 120 h to ensure equilibrium. After equilibrium, the supernatant from DI water samples was separated by centrifugation and filtered through a 0.2 μm PVDF syringe filter (Pall Corporation, Port Washington, NY, USA) prior to LC-MS/MS (LC-MS, IMAC, College Station, Texas) analysis.

Based on the pH-dependent adsorption results, AF900 was found to be the optimal biochar while pH 3.0 was identified as the optimal solution pH. An adsorption kinetic study was then performed at pH 3.0 and 23 °C by stirring 40 mL of PFOA and PFBA solutions (100 μg/L) with 10 mg of AF900 in 50 mL centrifuge tubes. Aliquots were withdrawn at predetermined time intervals and analyzed to evaluate adsorption kinetics. Adsorption isotherm experiments were carried out at pH 3.0 and 23 °C using PFOA and PFBA solutions with initial concentrations ranging from 25 to 2000 μg/L. For each experiment, 40 mL of solution was mixed with 10 mg of AF900 and agitated until equilibrium was reached. Thermodynamic experiments were performed in 100 mL of 50 mg/L PFOA or PFBA solution at pH 3.0 using 200 mg of AF900, and the adsorption equilibrium was evaluated at 293, 303, 313, and 323 K.

In this study, six kinetic models and four isotherm models were applied to fit the experimental data, and their fitting performances were evaluated using the coefficient of determination (R^2^). Kinetic, isotherm, and thermodynamic parameters were calculated using standard equations, and the relevant equations are summarized in Table S1.

To evaluate the competitive inhibitory effects between long-chain and short-chain PFAS, a binary batch system (PFOA–PFBA, both at 100 μg/L) was prepared at pH 3.0 and 23 °C. AF900 (10 mg) was added to 40 mL of the mixed solution and agitated until equilibrium.

Additionally, the reusability of AF900 was evaluated by performing cyclic adsorption-regeneration-readsorption to see potential reusability of AF900. First, 0.1 g of AF900 was fully saturated with PFOA (50 mL of 50 mg L^–1^ PFOA) or PFBA (50 mL of 25 mg L^–1^ PFBA) at pH 3.0 and 25 °C for 48 h until reaching equilibrium. After the adsorption, PFOA- and PFBA-saturated AF900 were separated and transferred to 50 mL of a 0.1% NH_3_·H_2_O methanol solution to regenerate AF900. The suspension was stirred for 48 h to promote desorption of PFOA and PFBA from the BC. Following the regeneration, AF900 was separated and reused to readsorb PFOA and PFBA under the same conditions as the initial adsorption step. The adsorption–regeneration–readsorption sequence was repeated for three cycles to evaluate the potential reusability of AF900.

### 2.5 Application of Biochars to PFAS-Contaminated Groundwater

To evaluate the adsorption performance of AF900 under environmentally relevant conditions, groundwater was used as the aqueous matrix (Table S2). The groundwater was collected from Stephenville, TX, USA. In the groundwater experiments, PFOA and PFBA were spiked to an initial concentration of 100 μg/L. In addition, the dosage of AF900 was varied (10, 50, 100, and 150 mg) to investigate the adsorption behavior of PFOA and PFBA under realistic environmental conditions without pH adjustment. After equilibrium, the supernatant was separated by centrifugation, and the remaining concentrations of PFOA and PFBA were analyzed after solid-phase extraction using a cartridge (Waters, Milford, MA, USA). The extracted analytes were redissolved in methanol and quantified by LC-MS/MS at the Core lab facilities at Texas A and M University (College Station, Texas, USA).

## 3 Results and Discussion

### 3.1 Characterization of Adsorbents

The physicochemical properties of alfalfa-derived biochars produced at different pyrolysis temperatures (300–900 °C) were systematically characterized using ultimate analysis, proximate analysis, BET surface and pore structure analysis, FT-IR spectroscopy, X-ray photoelectron spectroscopy (XPS), and X-ray diffraction (XRD). The results showed that pyrolysis temperature affected the elemental composition, pore development, surface functionality, and carbon structure of the AF-BCs (Table 1, Figures 1 and 2).

**Figure 1:**
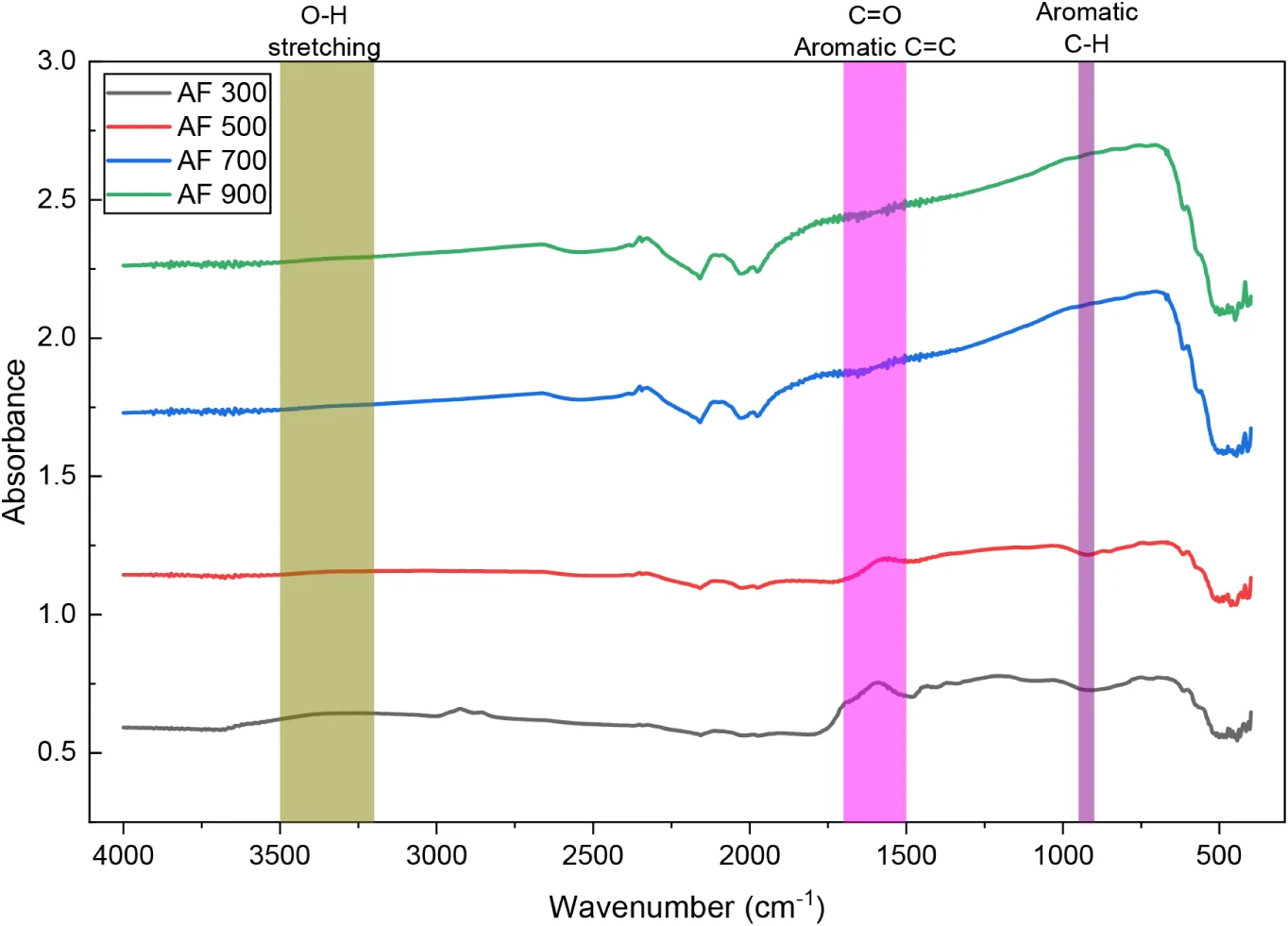
FTIR spectra showing surface functional groups of alfalfa (AF)-derived biochars made at various pyrolysis temperature. AF300–AF900 represent alfalfa-derived biochars labeled according to their pyrolysis temperatures (300, 500, 700, and 900 °C).

**Table 1:** Physical and Chemical Properties of Alfalfa-Derived Biochars^a^

	Ultimate analysis (%)				O/C and H/C (mol/mol)		Proximate analysis (%)			Pore volume and BET surface analysis (m^2^/g)			
Adsorbents	C	H	N	O	O/C	H/C	Volatile carbon	Fixed carbon	Ash	Micro, <2 nm	Meso, 2–50 nm	Macro, >50 nm	Specific surface area
AF300	64.26 ± 0.10	4.93 ± 0.05	4.83 ± 0.10	20.08 ± 0.12	0.235	0.915	74.3 ± 4.1	19.8 ± 4.3	5.9 ± 0.2	0.17 ± 0.03	2.45 ± 0.71	0.87 ± 0.16	4.80
AF500	64.44 ± 0.61	2.42 ± 0.22	3.59 ± 0.04	17.02 ± 0.97	0.198	0.448	56.4 ± 2.4	31.2 ± 1.7	12.4 ± 0.7	0.84 ± 0.13	9.78 ± 0.15	0.64 ± 0.02	46.69
AF700	65.54 ± 0.41	1.46 ± 0.03	3.55 ± 0.31	16.40 ± 0.50	0.188	0.265	46.9 ± 5.3	40.1 ± 4.4	13.0 ± 0.9	1.96 ± 0.02	21.55 ± 0.61	1.74 ± 0.78	186.54
AF900	66.16 ± 1.14	0.97 ± 0.03	1.65 ± 0.04	16.77 ± 1.12	0.190	0.174	36.8 ± 0.6	48.8 ± 1.8	14.4 ± 1.2	2.28 ± 0.19	22.89 ± 2.94	1.36 ± 0.47	242.29

a AF300–AF900 represent alfalfa-derived biochars labeled according to their pyrolysis temperatures (300, 500, 700, and 900 °C).

**Figure 2:**
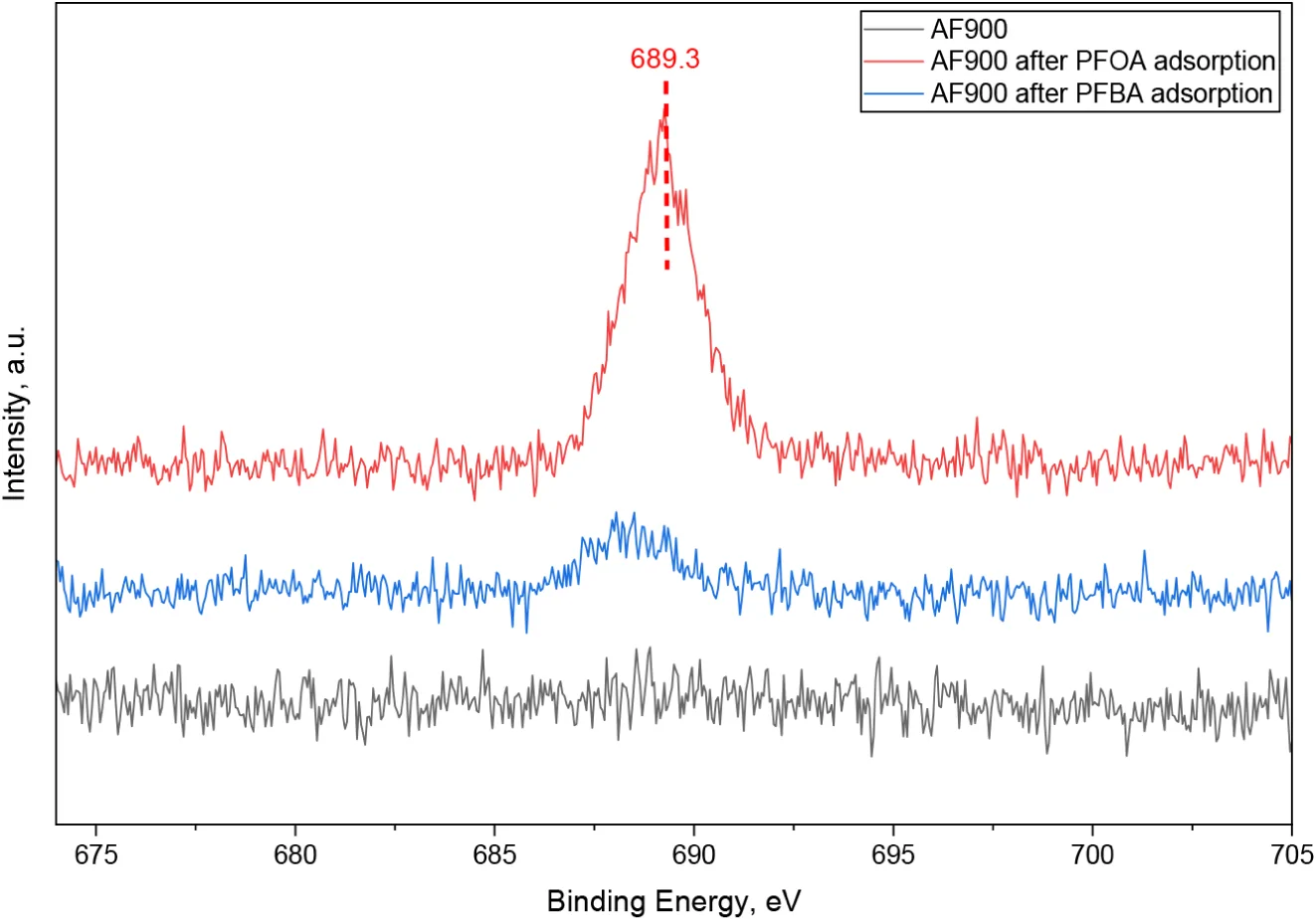
High-resolution F 1s XPS spectra of AF900 before and after PFOA and PFBA adsorption.

Ultimate analysis showed that increasing pyrolysis temperature resulted in a gradual enrichment of carbon content, increasing from 64.26% in AF300 to 66.16% in AF900. Hydrogen and nitrogen contents, however, decreased as the pyrolysis temperature increased. The hydrogen content decreased from 4.93% in AF300 to 0.97% in AF900, which is consistent with dehydrogenation and condensation during pyrolysis.^31^ This decrease was accompanied by greater carbonization and aromatization at higher temperatures.

The concurrent decreases in hydrogen and nitrogen contents provide further evidence of progressive carbonization during high-temperature pyrolysis. The decrease in hydrogen content, together with the lower H/C ratio (Table 1), reflects dehydrogenation and condensation of carbon, leading to a greater proportion of aromatic and nonpolar carbon domains. These structural changes can enhance hydrophobic interactions of PFOA and PFBA, particularly PFOA with its long-chain structure. The decrease in nitrogen content, from 4.83% in AF300 to 1.65% in AF900, also likely due to thermal decomposition and volatilization of nitrogen-containing compounds at higher pyrolysis temperature. This loss may reduce the abundance of polar or nitrogen-containing surface sites that could otherwise participate in hydrogen bonding or localized electrostatic interactions with polar functional groups of PFAS. Thus, while the decrease in hydrogen and nitrogen contents indicates increased carbon condensation and aromaticity that may favor nonpolar interactions, the simultaneous loss of polar nitrogen-containing sites may limit specific interactions with the more hydrophilic PFBA.

Consistent temperature-dependent behaviors were also observed in the proximate analysis. Volatile matter content decreased substantially from 74.3% in AF300 to 36.8% in AF900, reflecting extensive removal of thermally labile organic components. Conversely, fixed carbon content increased from 19.8% to 48.8%, indicating the formation of a more condensed and thermally stable carbon structure. Ash content increased from 5.9% to 14.4% with increasing temperature, which may be attributed to the relative enrichment of inorganic mineral components as organic matter volatilized.^32^ BET surface area analysis revealed a pronounced increase in specific surface area with increasing pyrolysis temperature: from the lowest surface area (4.80 m^2^/g) in AF300 to the highest surface area (242.29 m^2^/g) in AF900 (Table 1). Pore size analysis showed that micropores (<2 nm) were minimally developed at low temperature but increased steadily with temperature, while mesopores (2–50 nm) contributed most significantly to surface area development, increasing from 2.45 m^2^/g in AF300 to 22.89 m^2^/g in AF900. Macropores (>50 nm) increased from AF300 to AF700 but slightly decreased at AF900, suggesting partial pore restructuring or collapse at very high temperatures.^33^

FT-IR spectra further revealed systematic changes in surface functional groups with increasing pyrolysis temperature (Figure 1). The broad O–H stretching band in the 3200–3000 cm^–1^ region progressively weakened at higher temperatures, indicating thermal decomposition of hydroxyl-containing functional groups and dehydroxylation reactions.^34,35^ Meanwhile, the absorption intensity in the 1500–1700 cm^–1^ region decreased overall with increasing temperature, reflecting the loss of oxygen-containing functional groups, particularly carbonyl/carboxyl functionalities, with possible overlap from aromatic C=C vibrations.^36^ A weak absorption band at approximately 900–950 cm^–1^ was observed in AF300 and AF500 and was tentatively assigned to the out-of-plane bending vibration of aromatic C–H groups located at the edges of relatively small or less-condensed aromatic domains.^37^ The disappearance of this band in AF700 and AF900 is consistent with progressive dehydrogenation and condensation at higher pyrolysis temperatures, which reduced the abundance of peripheral aromatic C–H groups. Therefore, the loss of this band does not indicate decrease of aromaticity but rather the formation of more condensed and less hydrogenated aromatic carbon structures.

Additional FTIR spectra of AF900 before and after PFOA and PFBA adsorption were also analyzed (Figure S1). Compared with pristine AF900, PFOA- and PFBA-loaded AF900 showed noticeable disappearance mainly around 3800 cm^–1^ and 1600 cm^–1^ regions. It indicated possible interactions of PFOA and PFBA with isolated or weakly hydrogen-bonded surface O–H groups (around 3800 cm^–1^) as well as carbon–oxygen group (around 1600 cm^–1^) during adsorption.^38^ These changes suggest that surface functional groups of AF900 may have participated in PFAS adsorption through hydrogen bonding and electrostatic interactions.

The XRD patterns of AF-BCs showed broad diffraction features mainly in the 20–30° 2θ region, with no distinct sharp peaks corresponding to crystalline graphite (Figure S2). The overall XRD profiles were broadly similar among AF300–AF900, indicating that the biochars remained predominantly amorphous or turbostratic rather than highly graphitized.^39^ Although the XRD patterns did not show pronounced temperature-dependent differences, these results support the interpretation that the carbon structure was largely disordered. The predominantly amorphous carbon structure of AF-BCs may favor adsorption by providing diverse adsorption sites, including edge sites, defects, and irregularly stacked aromatic regions.^40,41^ Previous studies have also reported that amorphous porous biochars can favor adsorption through the combined effects of disordered carbon structures, developed porosity, and surface heterogeneity.^42,43^

As pyrolysis temperature increased, the O/C and H/C ratios decreased from 0.2346 to 0.1903 and from 0.9148 to 0.1741 (Figure S3), respectively, consistent with dehydration, decarboxylation, dehydroxylation, and progressive condensation of the carbon structure.^44^ The low O/C atomic ratio of AF900 indicates extensive deoxygenation during high-temperature pyrolysis and is consistent with a relatively low abundance of oxygen-containing surface functionalities. Please note that the O/C ratio represents the bulk elemental composition and cannot directly quantify acidic surface functional groups.

In addition, the previous thermal analysis of alfalfa feedstock (TG and DSC) showed that most mass loss occurred below 600 °C, with the maximum decomposition rate observed near 300 °C, while continued mass loss at higher temperatures was attributed mainly to further lignin decomposition (Figure S4).^23^ The corresponding DSC results also indicated a transition in thermal behavior at elevated temperatures, providing supporting context for the progressive removal of thermally labile components and further carbon condensation with increasing pyrolysis temperature. As reported by previous study,^17,33^ increasing pyrolysis temperature transformed AF-BC from volatile-rich, low-surface-area materials into more condensed carbonaceous solids with developed pore structures.

To further verify PFAS adsorption on the AF900 surface, XPS analysis was performed before and after PFAS adsorption (Figure 2 and Table S3). Pristine AF900 showed no detectable F signal, whereas PFOA-loaded AF900 exhibited a distinct F 1s peak centered, confirming the adsorption of fluorinated PFAS species onto the AF900 surface. In contrast, only a weak F 1s signal was observed for PFBA-loaded AF900, indicating substantially lower fluorine accumulation than that observed for PFOA-loaded AF900. These observations were consistent with the XPS surface elemental compositions (Table S3), where the surface fluorine content increased from not detected in pristine AF900 to 2.85 at. % after PFOA adsorption, whereas only 0.61 at. % was detected after PFBA adsorption. The substantially higher surface fluorine content after PFOA adsorption agrees well with the adsorption experiments, demonstrating the greater affinity of AF900 toward the longer-chain PFOA than PFBA.

### 3.2 Effect of pH on PFOA and PFBA Adsorption

The effects of solution pH on PFOA and PFBA adsorption by AF-BCs produced at different pyrolysis temperatures were investigated (Figure 3). Adsorption of PFOA and PFBA was highest at pH 3 and decreased progressively with increasing solution pH for all biochars, demonstrating pronounced pH-dependent adsorption behavior.^45^ AF900 achieved the highest adsorption capacities for PFOA (0.394 mg/g) and PFBA (0.097 mg/g) at pH 3. At pH 9, PFOA adsorption by AF900 decreased to 0.278 mg/g, whereas the calculated PFBA adsorption capacities were nearly zero for all biochars.

**Figure 3:**
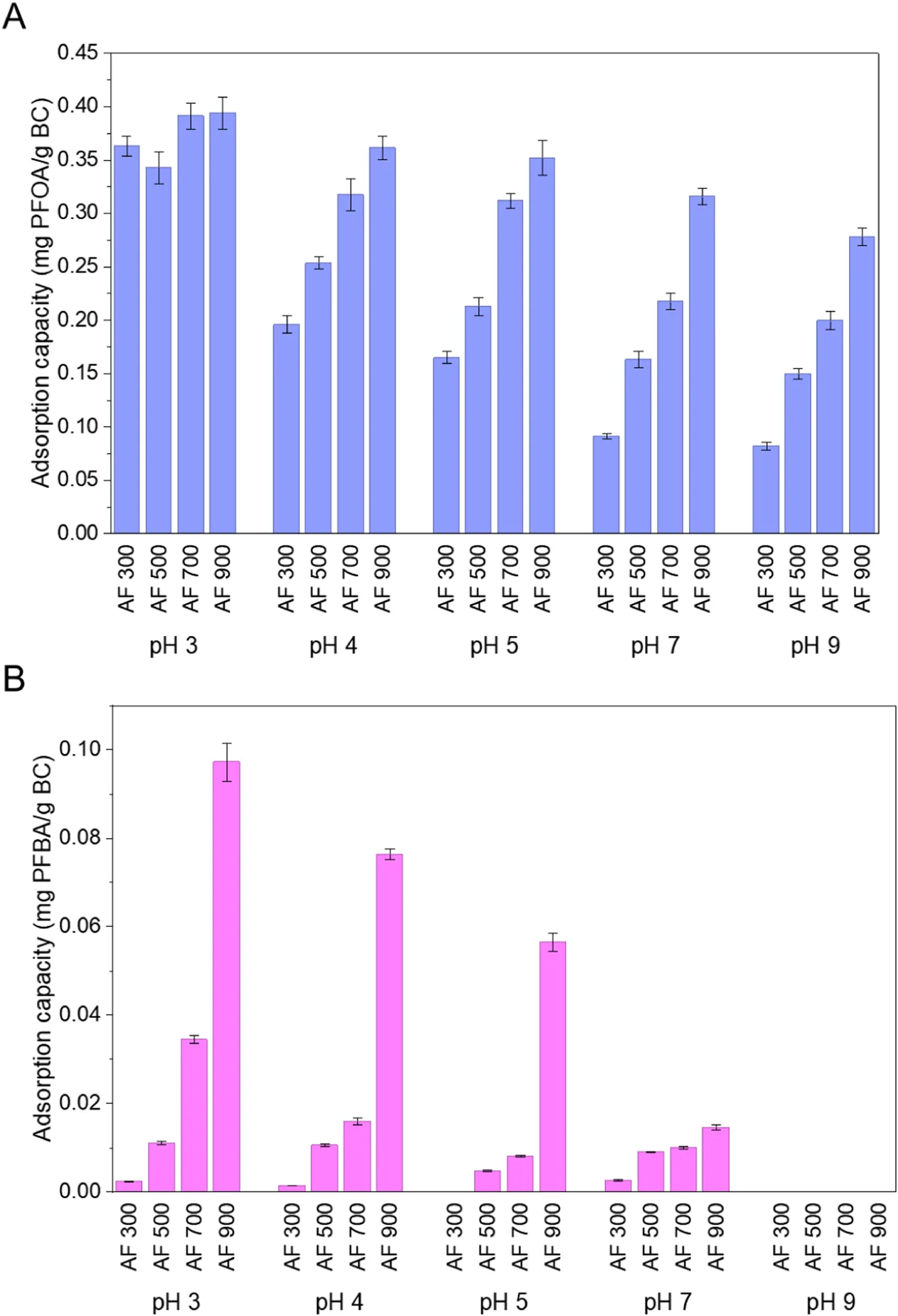
Adsorption of PFOA (A) and PFBA (B) on AF biochars at various pH. AF300–AF900 represent alfalfa-derived biochars labeled according to their pyrolysis temperatures (300, 500, 700, and 900 °C).

Consistent with the well-established chain-length dependence of PFAS adsorption, PFOA showed higher adsorption than PFBA across all biochars and pH conditions. This difference may be attributed to the shorter perfluoroalkyl chain length and lower hydrophobicity of PFBA, resulting in weaker affinity toward carbonaceous surfaces.^46^

The strong pH dependence observed for AF-BCs can be primarily attributed to pH-induced changes in biochar surface charge and interaction environments rather than changes in PFAS speciation. Both PFOA and PFBA are strong acids (p*K*_a_ < 1), and predominantly exist in their anionic forms over the investigated pH range.^47^ Since the experimentally determined pHpzc of AF900 was 6.21 (Figure S5), electrostatic attraction between the positively charged AF900 surface and anionic PFAS likely contributed substantially to adsorption at pH below the pHpzc, whereas increasing electrostatic repulsion at pH above the pHpzc contributed to the decline in adsorption with increasing pH (Table S4).

However, electrostatic interactions alone cannot explain the observed adsorption behaviors. PFOA retained substantial adsorption even at pH 9, where the AF900 surface was expected to be negatively charged, indicating that nonelectrostatic interactions also remained important under unfavorable electrostatic conditions. The greater adsorption of PFOA than PFBA suggests a higher contribution of hydrophobic association between the longer perfluoroalkyl chain of PFOA and the increasingly carbonized and less polar surface of AF900. In contrast, the lower adsorption and higher pH sensitivity of PFBA suggest that short-chain PFAS adsorption depended more heavily on favorable electrostatic conditions because of its lower hydrophobic affinity.

### 3.3 Correlation between Biochar Properties and PFOA/PFBA Adsorption

Pearson correlation analysis was performed to evaluate the relationships between the physicochemical properties of AF-BCs and their adsorption capacities for PFOA and PFBA (Table 2). Pyrolysis temperature, fixed carbon content, and specific surface area showed positive Pearson correlation coefficients with the adsorption of PFOA and PFBA, whereas volatile matter, O/C ratio, and H/C ratio showed negative coefficients. Among the examined variables, specific surface area showed the strongest positive correlation with PFOA adsorption (r = 0.868). For PFBA, pyrolysis temperature, fixed carbon content, and specific surface area showed relatively strong positive correlations (r = 0.928, 0.905, and 0.905, respectively), while volatile matter showed the strongest negative correlation (r = −0.871).

**Table 2:** Pearson Correlation Coefficients (r), Significance Levels, and p-Values for the Relationships between the Physicochemical Properties of Alfalfa-Derived Biochars and the Adsorption of PFOA and PFBA^a^

	PFOA adsorption			PFBA adsorption		
Factors	r	Significance level	p value	r	Significance level	p value
Temperature	0.749	NS	0.251	0.928	NS	0.0716
Volatile Carbon (%)	–0.657	NS	0.343	–0.871	NS	0.1290
Fixed Carbon (%)	0.716	NS	0.284	0.905	NS	0.0948
Surface area (m^2^/g)	0.868	NS	0.132	0.905	NS	0.0952
O/C (mol/mol)	–0.524	NS	0.476	–0.737	NS	0.2630
H/C (mol/mol)	–0.570	NS	0.430	–0.761	NS	0.2630

a *, **, and *** are significance levels of <0.05, <0.01 and <0.001; NS = not significant.

Although none of the Pearson correlations were statistically significant, the coefficients were interpreted only as descriptive trends and not as evidence of statistically demonstrated relationships between individual biochar properties and PFAS adsorption. Nevertheless, the directions of the coefficients were qualitatively consistent with previously reported pyrolysis-temperature-dependent changes in biochar properties, including pore development, carbon condensation, reduced volatile matter, and lower H/C and O/C ratios. These qualitative relationships also agree with recent PFAS adsorption studies showing that adsorption by carbonaceous sorbents depends on the combined effects of pore structure, specific surface area, surface chemistry, carbon structure, and hydrophobicity of PFAS with chain length.^48,49^

In this study, the specific surface area increased by approximately 50-fold, from 4.80 m^2^/g for AF300 to 242.29 m^2^/g for AF900, concurrently with the improved adsorption of both PFOA and PFBA. The positive coefficients between specific surface area and adsorption capacity for PFOA (r = 0.868) and PFBA (r = 0.905) were therefore considered qualitatively consistent with a contribution from increased accessible surface area and pores.

However, the substantially lower adsorption of PFBA than PFOA on AF900, despite their exposure to the same high surface area, demonstrates that specific surface area alone could not determine adsorption performance. A high surface area increases the number of potentially available adsorption sites, but effective adsorption also depends on the affinity of the PFAS molecule for those sites. The higher hydrophobic affinity of PFOA enabled it to interact more effectively with the nonpolar and condensed carbon domains of AF900, whereas the lower hydrophobicity and higher aqueous-phase hydration of PFBA limited its retention. Therefore, specific surface area can be interpreted along with other parameters to understand adsorption of PFAS. The positive coefficients for fixed carbon content and the negative coefficients for O/C and H/C ratios were also qualitatively consistent with the concurrent development of pores, increased carbon condensation and aromaticity, and reduced surface polarity reported for biochars produced at higher pyrolysis temperatures.^50,51^ Because these physicochemical properties changed concurrently with pyrolysis temperature, their individual contributions and relative importance cannot be quantitatively distinguished from the present correlation analysis.

### 3.4 Adsorption Kinetics, Isotherms, Thermodynamics, and Reusability

Kinetic, equilibrium isotherm, and thermodynamic analyses as well as reusability were performed for PFOA and PFBA adsorption onto AF900. AF900 at pH 3.0 was used for these experiments based on the pH-dependent adsorption results described in Section 3.2.

#### 3.4.1 Adsorption Kinetics

Both PFOA and PFBA were rapidly adsorbed during the initial stage, followed by slower uptake as equilibrium was approached (Table 3 and Figure S6). The decrease in adsorption rate with time was associated with progressive occupation of the available adsorption sites.

**Table 3:** Kinetic and Isotherm Model Parameters for the Adsorption of PFOA and PFBA onto AF 900 at pH 3.0 under Single-Solute Conditions

**Kinetic model**	**Parameter**	**PFOA**	**PFBA**	**Isotherm model**	**Parameter**	**PFOA**	**PFBA**
Pseudo-first-order	Q_e_	0.34746	0.0672	Freundlich	K_f_	0.05998	0.01579
	K_1_	28.34875	2.63239		n_f_	1.64320	2.11164
	R^2^	0.90538	0.85461		R^2^	0.99638	0.95357
Pseudo-second-order	Q_e_	0.35675	0.07053	Langmuir	Q_m_	6.36929	0.20883
	K_2_	141.88725	53.58744		K_L_	0.00162	–1.9794 × 10^45^
	R^2^	0.96545	0.92017		R^2^	0.98408	–1.55317 × 10^–7^
Elovich	Q_e_ ^a^	0.39848	0.0828	Temkin	K_T_	0.25213	0.03959
	a	785407	2.52754		b_T_	0.48811	0.11462
	b	56.18956	127.60913		R^2^	0.72998	0.96266
	R^2^	0.95566	0.98442	Dubinin–Radushkevich	K_DR_	3.53688	0.43557
Intraparticle diffusion	K_i_	0.01599	0.00605		B_D_	0.02296	0.01790
	C_i_	0.25499	0.02920		R^2^	0.84962	0.86150
	R^2^	0.30776	0.68659				
Liquid film diffusion	K_fd_	0.00726	0.00101				
	R^2^	–5.32303	–1.10347				
Two-compartment first-order	F_fast_	0.33163	0.05747				
	K_fast_	33.84333	4.28852				
	K_slow_	8.20924 × 10^–4^	2.55929 × 10^–4^				
	R^2^	0.93355	0.92789				

a * is the calculated value.

Among the various kinetic models, the pseudo-second-order (PSO) model showed the best fit followed by the Elovich model for adsorption of PFOA onto AFF900 which were similar to those reported by previous studies.^52^ For PFBA, the Elovich model provided the best fit, exceeding the pseudo-second-order and two-compartment first-order models.

For PFOA, the equilibrium adsorption capacity estimated by the PSO model (0.35675 mg/g) was closer to the experimental value (0.37525 mg/g) than those estimated by the pseudo-first-order (PFO, 0.34746 mg/g) and Elovich (0.39848 mg/g) models. In contrast, for PFBA, the Elovich model provided the closest estimated value (0.08278 mg/g) to the experimental value (0.08059 mg/g), compared with the PFO (0.06720 mg/g) and PSO (0.07053 mg/g) models. The kinetic data were also well described by the TCFO model. The K_fast_ values (33.84 for PFOA and 4.28 for PFBA) were substantially higher than the corresponding K_slow_ values (0.00082 for PFOA and 0.00026 for PFBA), indicating a rapid initial adsorption stage followed by a much slower uptake stage.

In contrast, the intraparticle diffusion and liquid film diffusion models showed poor fits for both PFOA and PFBA, suggesting that neither intraparticle diffusion nor liquid film diffusion alone adequately described the overall adsorption rate. Therefore, the adsorption kinetics of both PFOA and PFBA onto AF900 were likely influenced by multiple processes rather than by a single rate-limiting diffusion step.^53^ From a mechanistic perspective, the rapid initial adsorption stage is associated with uptake at readily accessible external surfaces and developed mesoporous regions of AF900. The subsequent slower stage may reflect progressive occupation of lower-affinity sites and transport toward less accessible pore regions. The good fit to the Elovich model, particularly for PFBA, further indicates that adsorption occurred across sites with different apparent adsorption energies, consistent with the chemically and structurally heterogeneous surface of AF900. While considering the properties of AF900 and pH-dependent results, the kinetic behaviors could suggest that pore accessibility would control the temporal progression of uptake, whereas electrostatic, hydrophobic, and aromatic-domain interactions influenced the affinity of PFOA and PFBA for the available sites.

#### 3.4.2 Adsorption Isotherms

The adsorption isotherms of PFOA and PFBA onto AF900 at pH 3.0 were investigated. For PFOA, the Freundlich model provided the best fit to the experimental data, indicating adsorption on an energetically heterogeneous surface with a nonuniform distribution of adsorption energies (Figure S7 and Table 3).^54^ While the Langmuir model followed the Freundlich model, the Temkin and Dubinin–Radushkevich models showed poor fit to the experimental results. For PFBA, the Temkin model provided a good fit to the experimental data, suggesting that the heat of adsorption changed with increasing surface coverage, consistent with the assumptions of the Temkin model.^55^ The maximum adsorption capacities of PFOA and PFBA onto AF900 were estimated to be 6.369 and 0.436 mg/g, respectively. Table S5 summarizes the carbon source precursor, adsorbent category or modification, specific surface area, adsorbent dosage, initial PFAS concentration range, and maximum adsorption capacity reported in previous studies. Although the adsorption capacities of these carbon materials reported in Table S5 were obtained under different experimental conditions, the maximum adsorption capacities of the carbon materials and AF900 could indicate relative effectiveness of AF900 over other carbon materials. A detailed comparison of AF900 and commercially available carbon adsorbents, such as activated carbon and carbon nanotube, will be conducted as one of further works following this study. The maximum adsorption capacity of AF900 for PFOA was lower than those reported for granular activated carbon and activated biochars, but comparable to or higher than those of several pristine biochars (Table S5). The higher adsorption capacities of the activated carbon and biochars are likely attributable to additional physical or chemical activation, surface modification, and the resulting increases in specific surface area and pore development. In contrast, AF900 was produced solely through pyrolysis without further activation or post-treatment, demonstrating that nonactivated AF-BC can provide adsorption performance comparable to that of several pristine biochars. For PFBA, however, AF900 exhibited substantially lower adsorption capacities than several pristine and activated biochars, suggesting that pyrolysis alone without any modification would be insufficient to overcome the low affinity of short-chain PFAS onto surface of AF900.

The adsorption capacity of PFOA was substantially higher than that of PFBA across the equilibrium concentration range (Figure S7), indicating a stronger affinity of AF900 toward PFOA. The Freundlich fit for PFOA adsorption on heterogeneous sites, including external carbon surfaces, various pore regions, and condensed aromatic carbon structures. The stronger hydrophobic affinity of PFOA may increase its adsorption onto these nonpolar and aromatic structures, along with pore filling at increasing equilibrium concentrations.

In contrast, the Temkin model provided the best fit for PFBA, suggesting that its adsorption energy changed with increasing surface coverage. Due to its weaker hydrophobic affinity, PFBA adsorption may initially occur at a limited number of favorable polar or positively charged sites, followed by adsorption at lower-affinity sites. This may explain its relatively low adsorption capacity and strong dependence on aqueous-phase hydration. While the isotherm models alone cannot identify specific adsorption mechanisms, their interpretation together with pH dependence, biochar characterization, and kinetic results can provide insights into the possible adsorption mechanisms.^56,57^

#### 3.4.3 Adsorption Thermodynamics

The thermodynamic parameters for the adsorption of PFOA and PFBA onto AF900 are summarized in Table 4. For both compounds, the *K_d_* values increased with increasing temperature, indicating that adsorption became more favorable at elevated temperatures. Consistently, the positive Δ*H*° values obtained from the van’t Hoff plots confirmed that the adsorption of both PFOA and PFBA onto AF900 was endothermic.^58^ In addition, the positive Δ*S*° values suggest increased randomness at the solid–liquid interface during adsorption, which may be associated with the release of water molecules from the hydration shells of PFAS molecules or the adsorbent surface during interfacial interaction.^59,60^

**Table 4:** Thermodynamics Parameters for the Adsorption of PFOA and PFBA onto AF900 at pH 3.0

Adsorbate	Temperature (K)	*K*_d_ (L/g)	ΔG^0^ (kJ/mol)	ΔH^0^ (kJ/mol)	ΔS^0^ (J/mol·K)
PFOA	293.15	0.13	5.03	75.36	240.23
	303.15	0.32	2.91		
	313.15	1.46	–0.98		
	323.15	1.82	–1.61		
PFBA	293.15	0.004	13.24	82.76	238.55
	303.15	0.016	10.37		
	313.15	0.074	6.76		
	323.15	0.085	6.61		

Despite these common trends, the thermodynamic behavior of PFOA and PFBA differed markedly. For PFOA, the *K_d_* value increased from 0.13 L/g at 293.15 K to 1.82 L/g at 323.15 K, and the Δ*G*° values changed from positive (5.04 kJ/mol) at 293.15 to negative (−0.98 and −1.61 kJ/mol) at 313.15 and 323.15 K. This indicates that PFOA adsorption was less favorable at lower temperatures but became thermodynamically more favorable at elevated temperatures. The positive Δ*H*° value was consistent with the observed increase in PFOA adsorption at higher temperatures. PFBA also showed an increase in *K_d_* with temperature, but its *K_d_* values remained lower than those of PFOA, and Δ*G*° remained positive throughout 293.15–323.15 K.

This indicates that PFBA adsorption was enhanced by increasing temperature, but did not become thermodynamically favorable within the investigated temperature range. Overall, AF900 exhibited more favorable thermodynamic behavior toward PFOA than toward PFBA under the same adsorption conditions.

#### 3.4.4 Reusability of Alfalfa-Derived Biochar with Chemical Regeneration

The regeneration of PFOA-saturated AF900 using 0.1% (v/v) NH_4_OH in methanol achieved 86% of the initial PFOA adsorption capacity after the first regeneration cycle, with the adsorption capacity gradually decreasing to 72% after the third cycle (Figure 4). In comparison, regeneration of PFBA-saturated AF900 using the same procedure retained 70–73% of the initial PFBA adsorption capacity during the first and second cycles, followed by a decline to 54% after the third cycle. The gradual decrease in regeneration efficiency over successive cycles was likely attributable to changes in the physicochemical properties of AF900, including possible alterations in surface chemistry, surface area, and pore structure caused by repeated solvent desorption, as has been reported for chemically regenerated carbonaceous adsorbents.^61^ Therefore, the chemical regeneration process for PFOA- and PFBA-saturated AF900 should be further investigated and optimized to improve its reusability and practical applicability. Nevertheless, although the regeneration protocol was not fully optimized in this study, the observed regeneration efficiencies demonstrate that AF900 has promising potential for reuse in the adsorption of both PFOA and PFBA.

**Figure 4:**
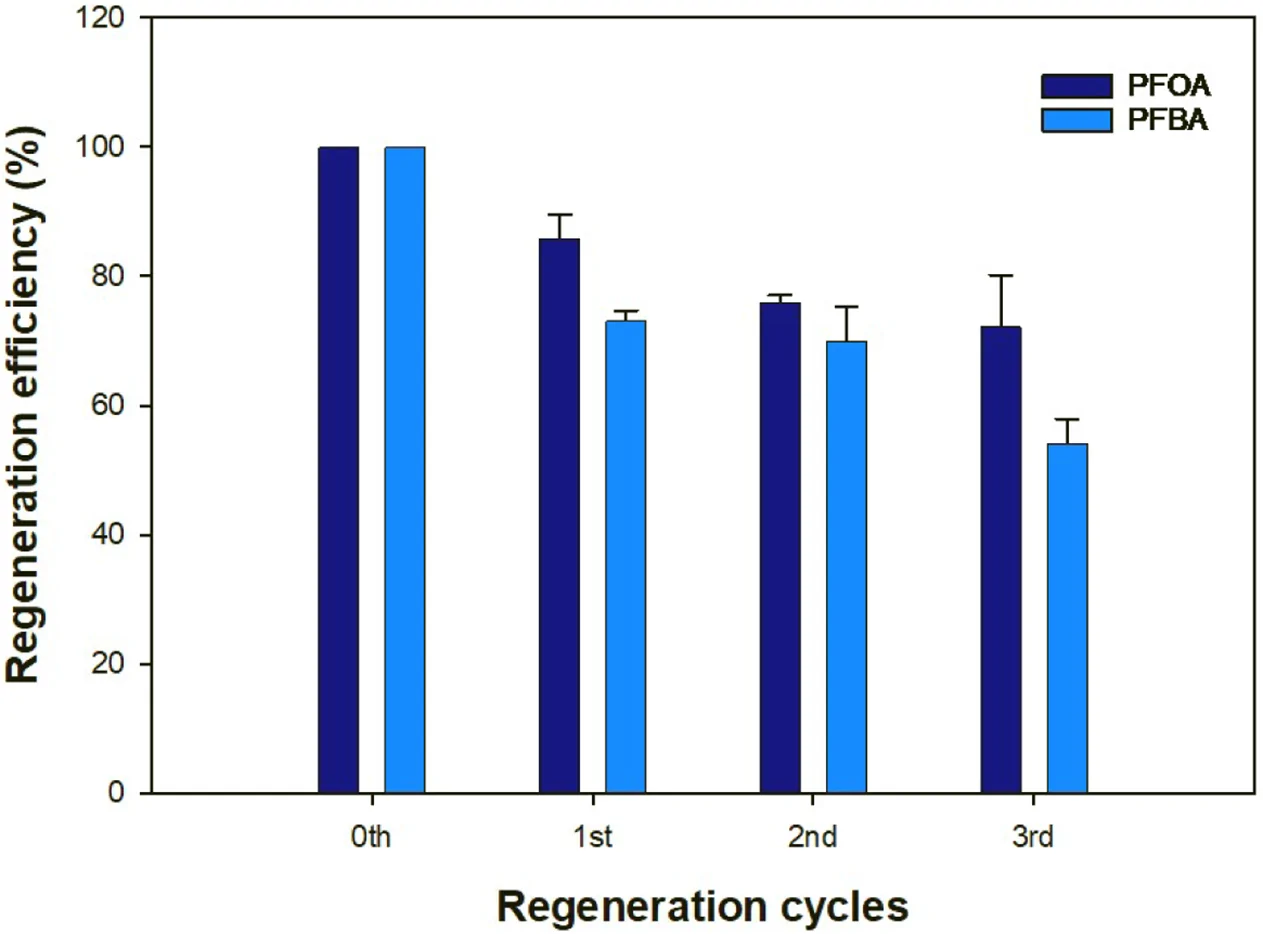
Reusability and chemical regeneration of AF-BC for PFOA and PFBA over three cycles. The o^th^ cycle indicates the initial adsorption of PFOA and PFBA onto AF-BC.

### 3.5 Competitive Inhibitory Effect of PFOA and PFBA in PFOA–PFBA Binary Solute System

To further evaluate competitive adsorption behavior, the adsorption kinetics of PFOA and PFBA were investigated in a PFOA–PFBA binary solute system and compared with those obtained from the corresponding single-solute experiments (Figure 5). The simultaneous adsorption of PFOA and PFBA in the PFOA–PFBA binary solute system resulted in only minor changes in adsorption behavior for both compounds, indicating that competitive inhibitory effects were limited under the investigated conditions. In the binary system, the adsorption profiles of both PFOA and PFBA were similar to those obtained in the corresponding single-solute experiments, including their behavior near equilibrium.

**Figure 5:**
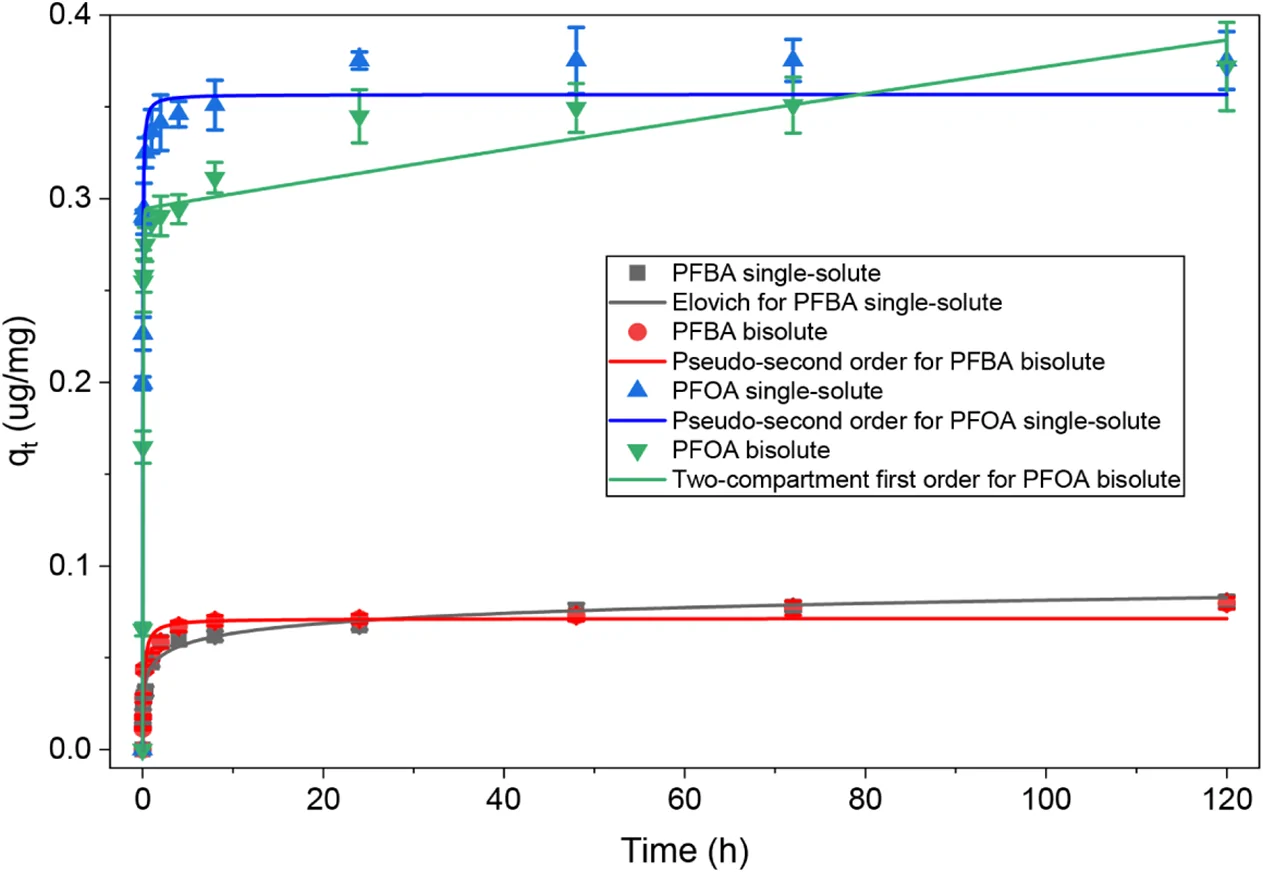
Adsorption kinetics of PFOA and PFBA on AF900 in single and binary solute systems at pH 3.0, along with the best-fitting kinetic models for each condition.

In the PFOA–PFBA binary system, PFOA showed faster and higher adsorption than PFBA, consistent with the chain-length-dependent affinity observed in the single-solute experiments and reported for other carbonaceous adsorbents.^21,62^ Importantly, the simultaneous presence of the two compounds caused only limited changes in their overall adsorption, indicating that competitive interference was relatively minor under the investigated conditions.

Kinetic model fitting differed between the single- and binary-solute systems (Figure S8 and Table S6). PFOA was best fitted by the PSO model in the single-solute system, whereas the two-compartment first-order model gave the best fit in the binary system. This difference indicates a clearer separation between the rapid and slow uptake stages under binary conditions, possibly reflecting adsorption at readily accessible sites followed by uptake at less accessible sites or pores. For PFBA, the Elovich model showed the best fit in the single-solute system. In the binary system, the PSO, Elovich, and two-compartment first-order models gave similar fits, although the PSO model was slightly better. This result indicates that PFBA adsorption became kinetically more mixed under binary conditions and more sensitive to site accessibility and site distribution. AF900 adsorbed both PFOA and PFBA in the binary system, with only a limited competitive effect between the two compounds. The differences in kinetic model fitting between the single- and binary-solute systems indicate changes in the relative contributions of the fast and slow uptake stages rather than a major change in the overall adsorption behavior.

### 3.6 Application of AF900 for Removal of PFOA and PFBA in Groundwater

To examine the adsorption behavior of AF900 in a more complex aqueous matrix, PFOA and PFBA adsorption were investigated using groundwater spiked to an initial concentration of 100 μg/L. The results were compared with those obtained in DI water listed in Section 3.2 (Figure 6A). Compared with DI water, adsorption in groundwater was substantially reduced, with removal efficiencies of 12.98% for PFOA and 2.81% for PFBA (Figure 6A). This reduced performance was likely associated with groundwater chemistry and coexisting constituents, which may affect surface interactions, PFAS transport, and the accessibility of adsorption sites (Table S2).

**Figure 6:**
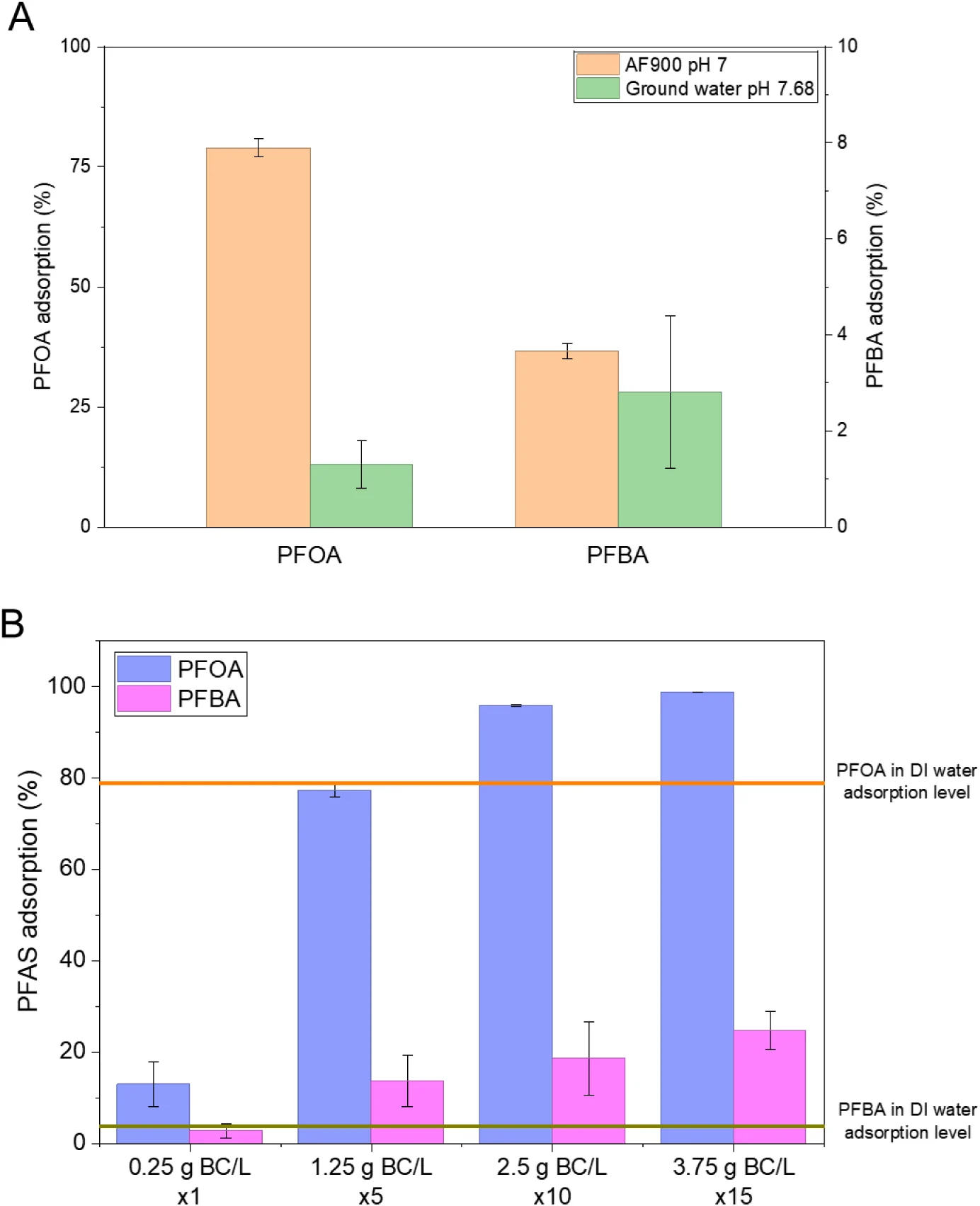
Adsorption capacities of PFOA and PFBA on AF900 under different conditions: (a) comparison of adsorption in DI water (pH 7.0) and groundwater (pH 7.68), and (b) effect of AF900 dosage (0.25–3.75 g BC/L) on adsorption in groundwater.

Increasing the AF900 dosage from 0.25 to 3.75 g/L markedly enhanced the removal of both PFOA and PFBA in groundwater (Figure 6B). PFOA removal increased from 12.98% at 0.25 g/L to 98.78% at 3.75 g/L. PFBA removal also increased with dosage, but the enhancement was more limited, increasing from 2.81% to 24.76% over the same biochar dosage range. These results reveal that increasing the dosage of AF900 improved PFOA removal despite the groundwater matrix effects, whereas PFBA removal remained comparatively limited. The limited improvement in PFBA removal, even at the high dosage of AF900, further indicates that increasing available adsorption sites alone could not compensate for its weak surface affinity, particularly in the presence of competing groundwater constituents.

The U.S. EPA has established an enforceable maximum contaminant level of 4.0 ng/L for PFOA in drinking water, whereas PFBA is not currently assigned an individual federal drinking-water MCL [61]. With the high AF900 dosage of 3.75 g/L, 98.78% PFOA removal from an initial concentration of 100 μg/L corresponded to a residual concentration of approximately 1.22 μg/L, which remained substantially above the US EPA standard for PFOA in drinking water. Therefore, although AF900 substantially reduced PFOA under the tested groundwater conditions, the adsorption of PFOA using AF900 would need further optimization and modification to achieve the drinking-water standard. For PFBA, the residual concentration at the highest dosage was approximately 75.24 μg/L, demonstrating that the unmodified AF900 was insufficient for effective removal of this short-chain PFAS under the tested conditions, requiring further biochar optimization and modification, additional adsorption stages, or integration with other treatment processes.

### 3.7 Possible Mechanisms Associated with Adsorption of PFOA and PFBA on AF900

The possible adsorption pathways are summarized conceptually in Figure 7, which is intended as an illustrative schematic and not as an actual morphological representation of AF900. PFOA and PFBA adsorption onto AF900 likely involved several concurrent interactions rather than a single dominant mechanism. At the pH below the pHpzc of AF900, electrostatic attraction between the positively charged biochar surface and the anionic groups of PFOA and PFBA promoted adsorption. The developed pore structure of AF900 also increased the number of accessible adsorption sites and allowed retention through pore filling. In addition, the condensed and relatively nonpolar carbon domains formed during high-temperature pyrolysis favored hydrophobic interactions with the perfluoroalkyl chains. These nonelectrostatic interactions were more pronounced for PFOA because of its longer perfluoroalkyl chain, explaining its substantial adsorption even under less favorable electrostatic conditions. In contrast, PFBA depended more strongly on favorable electrostatic sites because its shorter chain provided weaker hydrophobic affinity. Therefore, Figure 4 represents the combined contributions of surface charge, pore accessibility, carbon structural properties, and PFAS chain length to the different adsorption behaviors of PFOA and PFBA.

**Figure 7:**
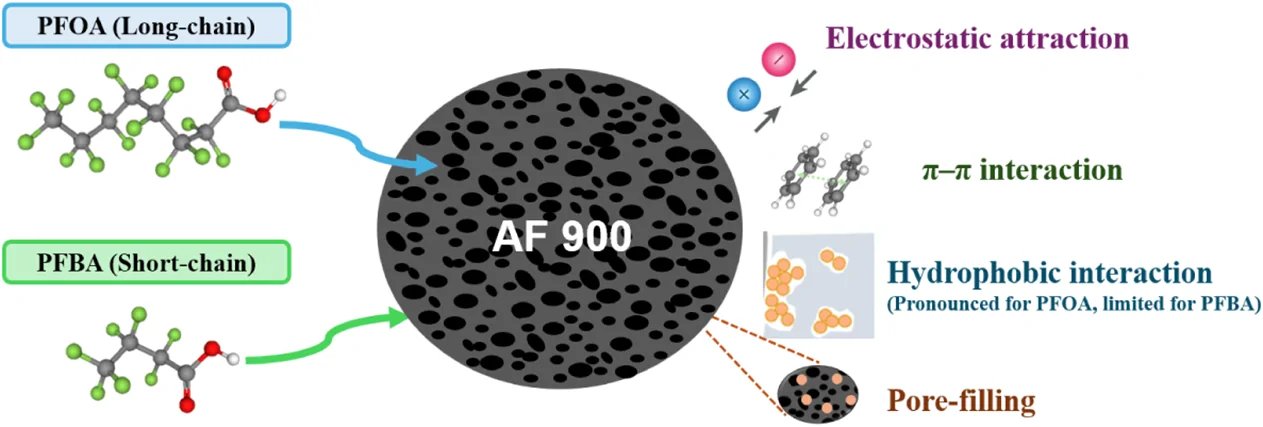
Conceptual schematic illustrating the possible mechanisms associated with the adsorption of PFOA and PFBA on AF900. The depicted biochar structure is an illustrative representation and does not represent the actual morphology of the AF900 sample.

Despite the presence of these potential nonhydrophobic adsorption pathways, they were insufficient to overcome the intrinsically weak affinity of PFBA for AF900. The shorter perfluoroalkyl chain of PFBA provides weaker hydrophobic interactions with the condensed carbon domains, while its relatively strong hydration in water can limit its transfer from the aqueous phase to the surface of AF900. Electrostatic attraction was favorable only at the solution pH below the pHpzc and weakened or became repulsive as the solution pH increased. In addition, high-temperature pyrolysis reduced the abundance of oxygen-containing functional groups that could otherwise contribute to specific interactions with the polar functional groups of PFBA. Therefore, although pore development increased the number and accessibility of potential adsorption sites, it did not provide sufficient affinity or selectivity for PFBA. This explains why AF900 improved PFBA adsorption relative to AF-BC produced at lower temperature, but remained substantially less effective for PFBA than for PFOA.

The possible mechanisms are supported by the properties of AF900, particularly porous structure, surface area, surface chemistry and functionalities listed in Section 3.1. AF900 possessed greater micro- and mesopore surface areas, providing more accessible adsorption domains and potential pore-filling sites. Simultaneously, the lower H/C and O/C ratios and the FTIR and XRD results indicated increased carbon condensation and aromaticity. These condensed aromatic carbon domains may provide favorable nonpolar and π-electron-rich environments for interaction with the fluorinated PFAS chains. Conversely, the decrease in oxygen-containing functional groups with increasing pyrolysis temperature, despite improved PFAS adsorption, suggests that polar surface functional groups were not the principal factor responsible for the enhanced adsorption, although residual functional groups may still contribute to localized interactions with PFAS. Thus, the enhanced adsorption by AF900 cannot be attributed to increased surface area alone but likely resulted from the combined effects of pore accessibility, carbon condensation and aromaticity, hydrophobic affinity, and pH-dependent electrostatic interactions.

## 4 Conclusions

This study demonstrated that pyrolysis temperature strongly influenced the physicochemical properties of alfalfa-derived biochar and its adsorption performance toward PFOA and PFBA. Among the alfalfa-derived biochars made at 300–900 °C, AF900 showed the best overall adsorption performance, and adsorption of both PFOA and PFBA was strongly pH-dependent, with the highest adsorption observed at pH 3.0. Under all tested conditions, PFOA consistently showed greater adsorption than PFBA. The adsorption kinetic, isotherm, and thermodynamic analyses revealed distinct adsorption behaviors of the two PFAS compounds, with PFOA better described by the pseudo-second order and Freundlich models, PFBA by the Elovich and Temkin models, and adsorption of both compounds being endothermic. The results also suggest that adsorption onto AF900 was likely governed by multiple mechanisms, including electrostatic attraction, hydrophobic interaction, interaction with condensed aromatic carbon domains, and pore filling. In the PFOA–PFBA binary solute system, competitive inhibition was limited, although coexisting PFAS slightly affected the apparent adsorption dynamics. AF900 showed a gradual decrease in regeneration efficiency over three cycles for PFOA and PFBA while relatively higher decrease for PFBA. Although the adsorption performance decreased in groundwater compared with DI water, increasing the biochar dosage improved adsorption, particularly for PFOA. However, the residual PFOA concentration remained above the U.S. EPA drinking-water standard even at the high AF900 dosage, while PFBA removal remained limited, indicating that further sorbent optimization and modification are required. Although AF900 provided multiple potential adsorption pathways, these mechanisms were insufficient to compensate for the weak hydrophobic affinity and strong aqueous-phase stabilization of PFBA, resulting in substantially lower and more matrix-sensitive removal than that of PFOA. Overall, AF900 was the best-performing material among the AF-BCs prepared in this study. Further work includes evaluation of AF900 over commercial activated carbon and carbon adsorbents for removal of various PFAS as well as additional modification of AF900 to enhance its adsorption capacities for long and short-chain PFAS in groundwater and wastewater.

## Supplementary Material



## Data Availability

The data supporting this study are available within the manuscript.

## Notes

Supplementary characterization results, adsorption model equations, groundwater quality, pH-dependent interaction analysis, comparison with previous biochar adsorbents, and additional kinetic and isotherm fitting results (PDF)
