# Supplementary material for: Tailoring Alfalfa-Derived Biochar via Pyrolysis Temperature for Enhanced Adsorption of Diverse Per- and Polyfluoroalkyl Substances in Water
Source: ACS Omega. 2026 Oct 22;11(43):66591–605. doi: 10.1021/acsomega.6c06097 (PMC13737923; doi:10.1021/acsomega.6c06097)
Supplement: Supplementary file 1 [file ao6c06097_si_001.pdf]

Tailoring Alfalfa-Derived Biochar via Pyrolysis Temperature for Enhanced Adsorption of Diverse Per- and Polyfluoroalkyl Substances in Water

Gi-Beom Kim[a,b], Soo Youn Lee[c], Sahng Hyuck Woo[d], Ying-Ren Lai[a,e], Eunsung Kan[a,e*]

[a]Texas A&M AgriLife Research and Extension Center, Stephenville, TX, 76401, USA

[b]Yonsei University, Seodaemun-gu, Seoul, 03722, Korea

[c]Gwangju Clean Energy Research Center, Korea Institute of Energy Research, Gwangju 61003, South Korea

[d]Jeju Global Research Center, Korea Institute of Energy Research (KIER), 200 Haemajihaean-ro, Gujwa-eup, Jeju-si, Jeju-do 63359, Republic of Korea

[e]Department of Biological and Agricultural Engineering, Texas A&M University, College Station, TX, 77843, USA

* Corresponding author. Tel: +1-254-968-4144 E-mail: eunsungkan@tamu.edu

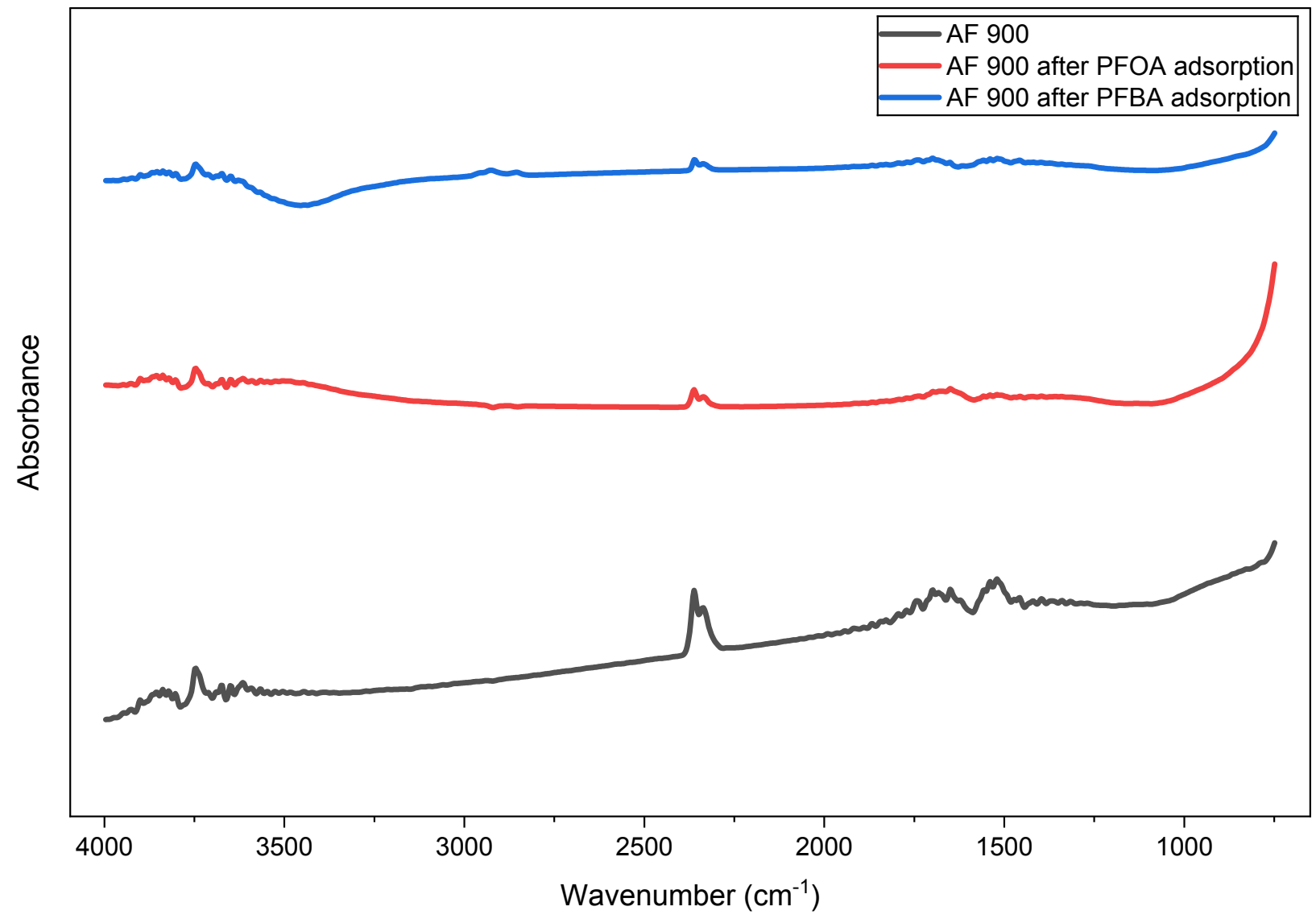

Figure S1. FTIR spectra of AF900 before and after PFOA and PFBA adsorption.

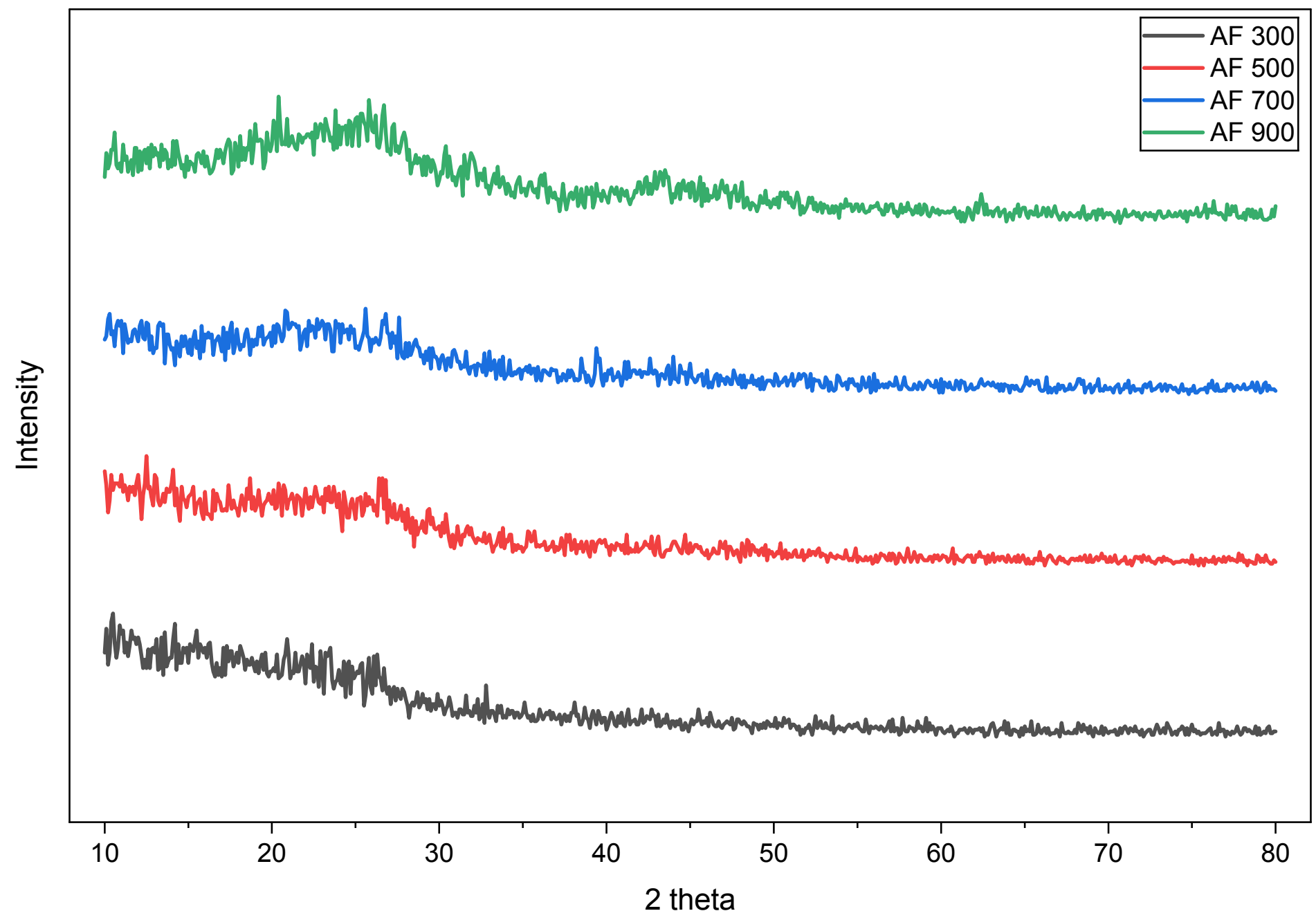

Figure S2. X-ray diffraction (XRD) patterns of AF biochars produced at different pyrolysis temperatures

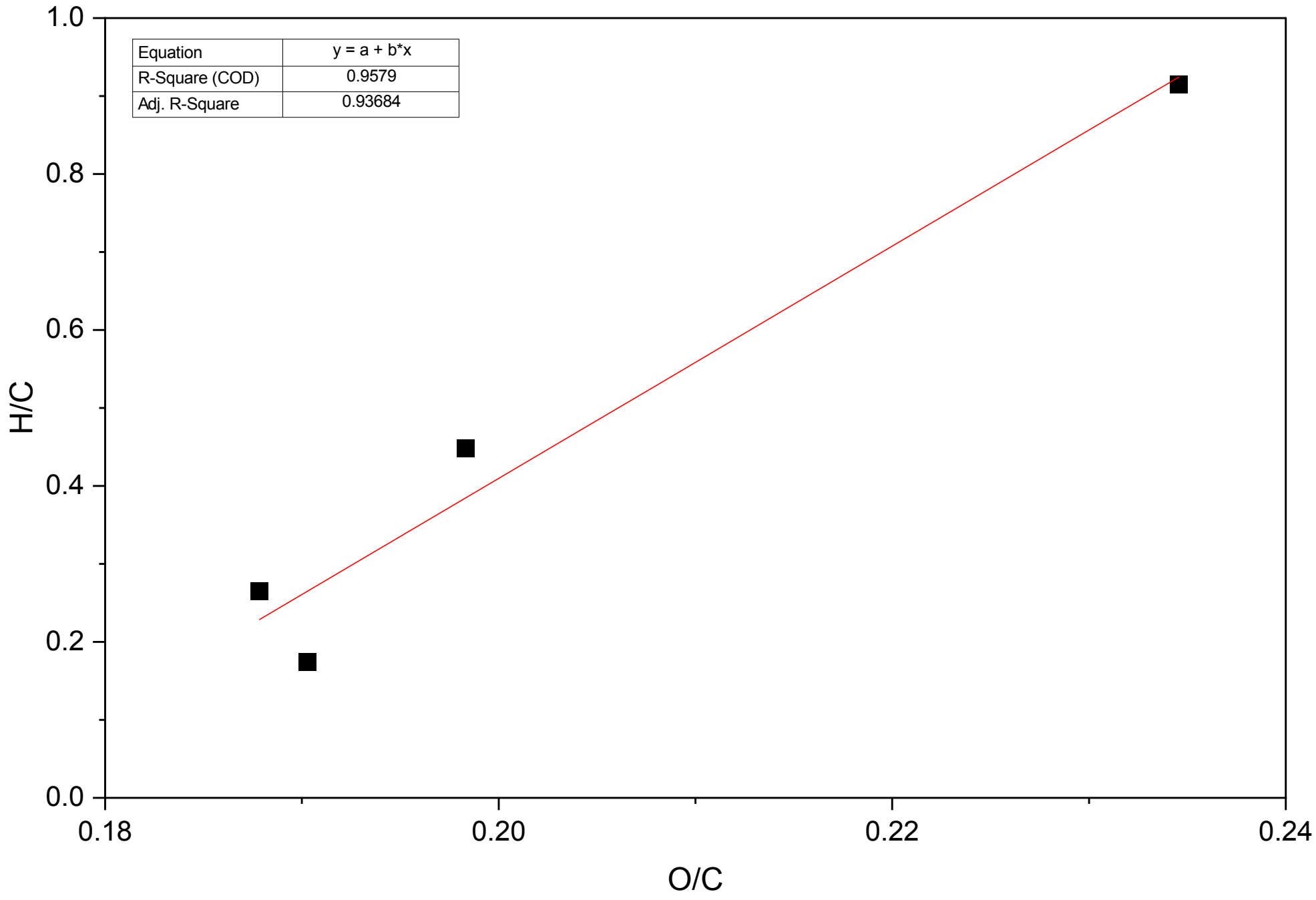

Figure S3. Van Krevelen diagram for relationships between O/C and H/C

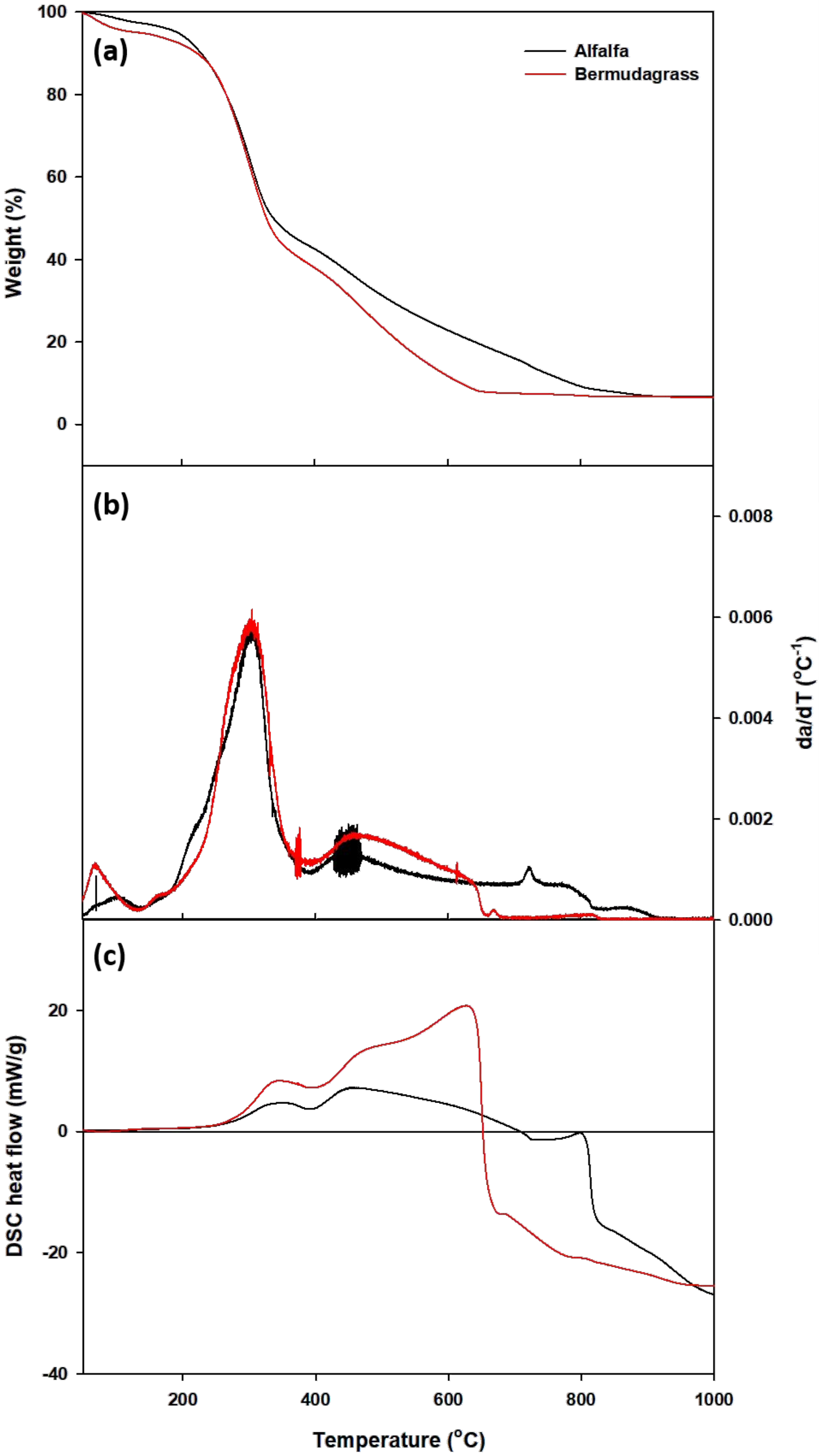

Figure S4. TG/DSC results of alfalfa and bermudagrass: Weight loss (a), reaction rate (b) and heat flow (c) versus temperature (°C) dependences (Jang and Kan, 2019) [1]

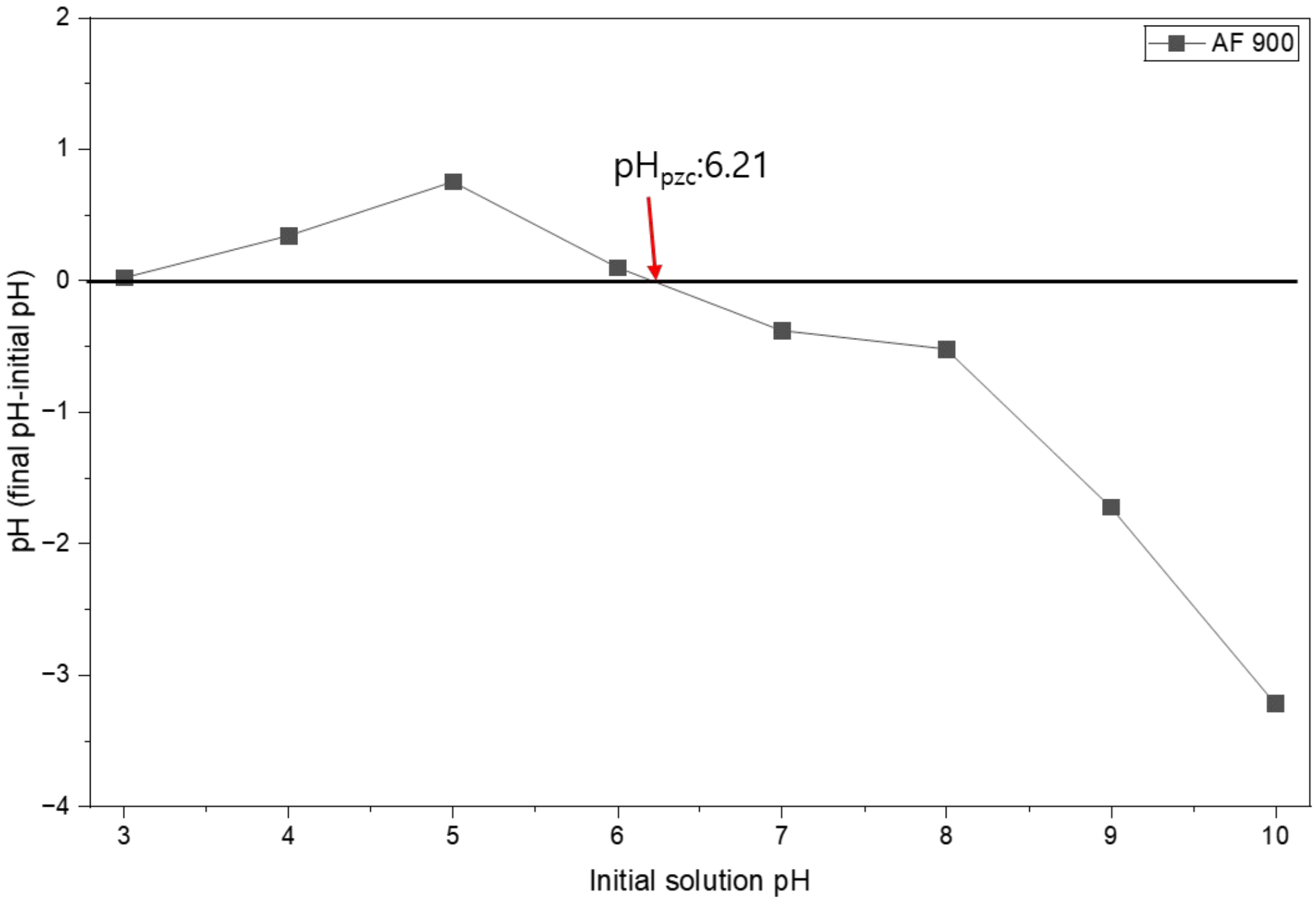

Figure S5. pH drift curve used to determine the pH_{pzc} of AF 900 biochar

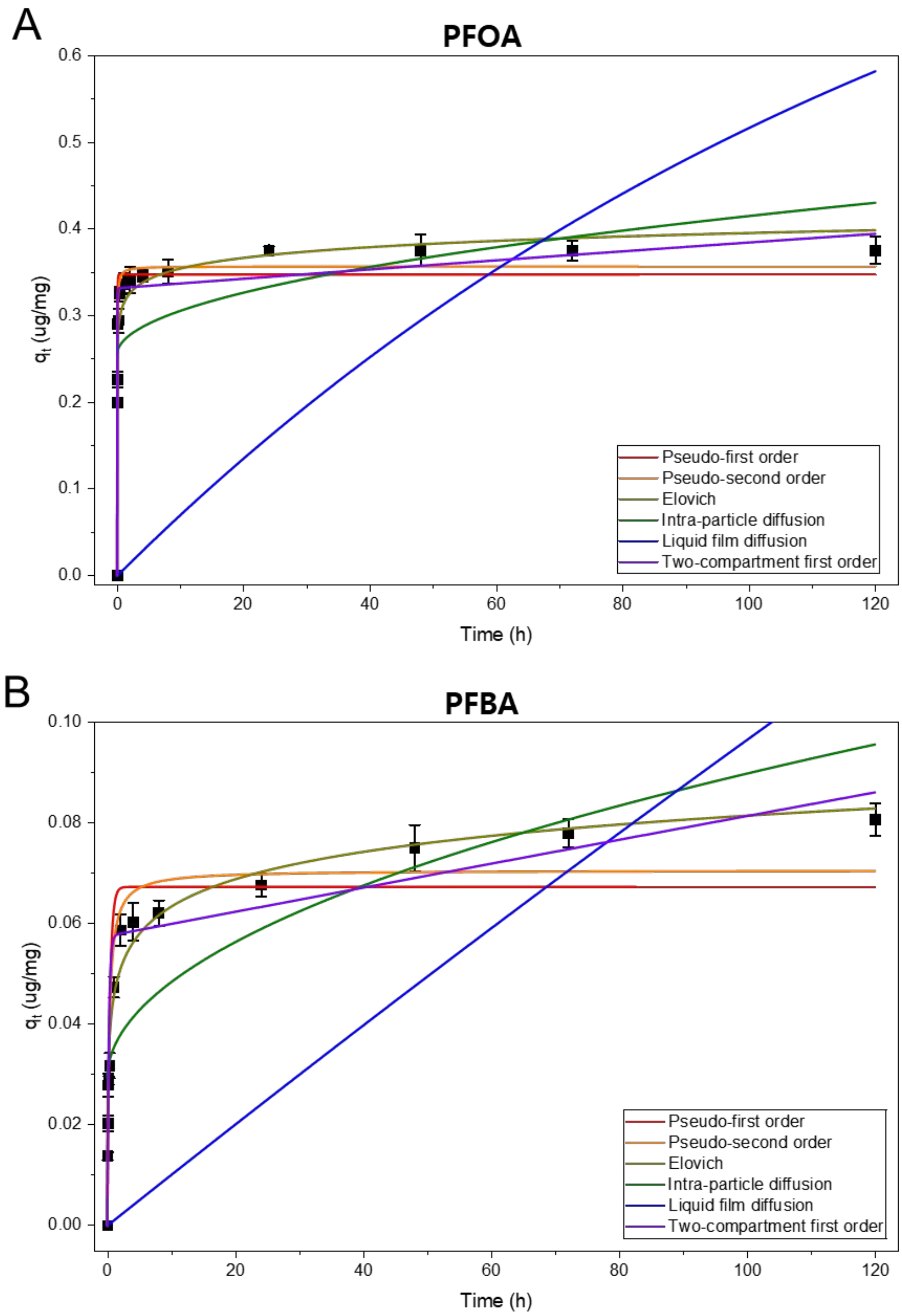

Figure S6. Experimental adsorption kinetics of PFOA (A) and PFBA (B) onto AF 900 at pH 3.0, together with the fitted curves of the pseudo-first-order, pseudo-second-order, Elovich, intraparticle diffusion, liquid film diffusion, and two-compartment first-order models.

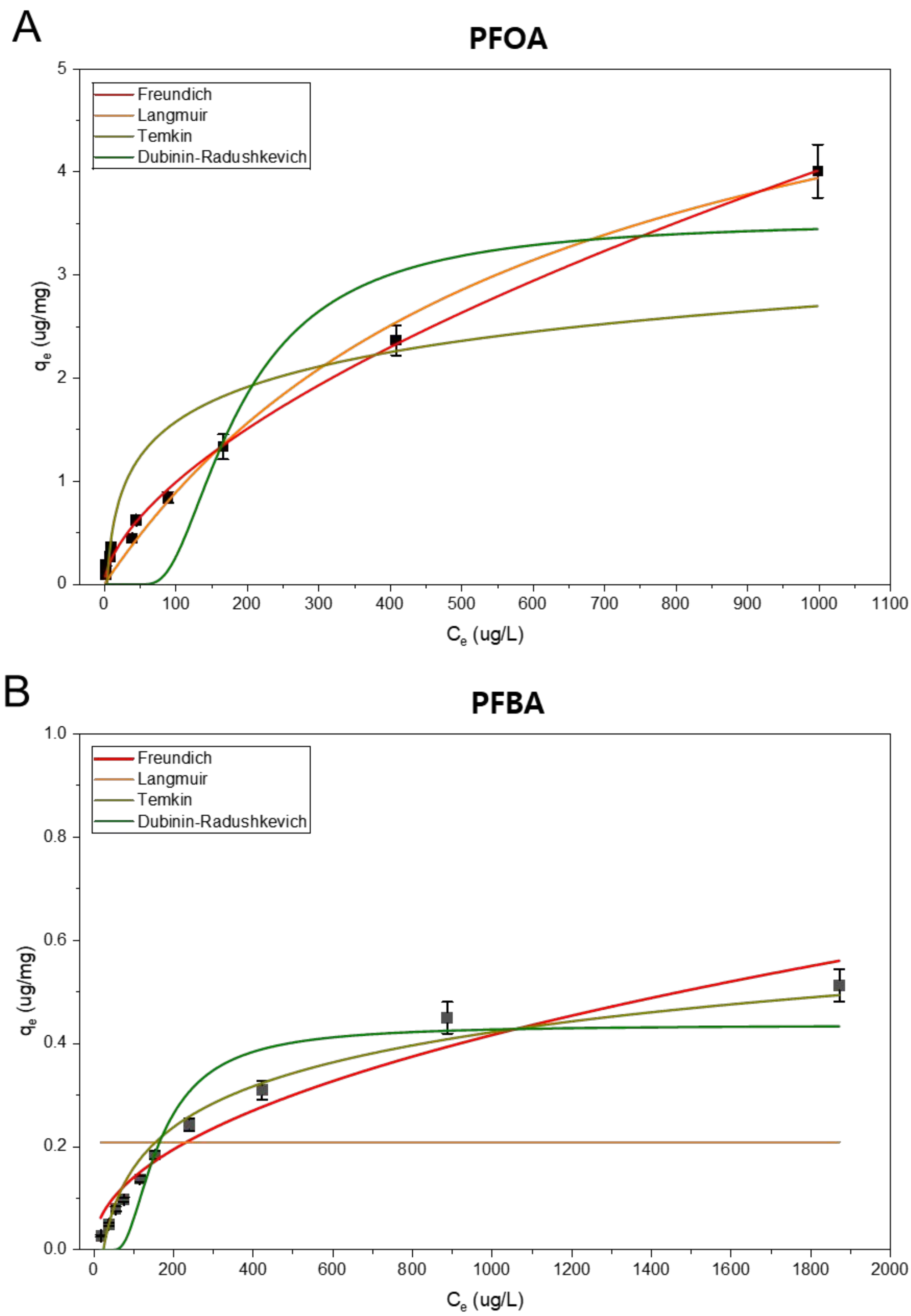

Figure S7. Experimental adsorption isotherms of PFOA (A) and PFBA (B) onto AF 900 at pH 3.0 and the corresponding fits obtained using the Freundlich, Langmuir, Temkin, and Dubinin–Radushkevich models.

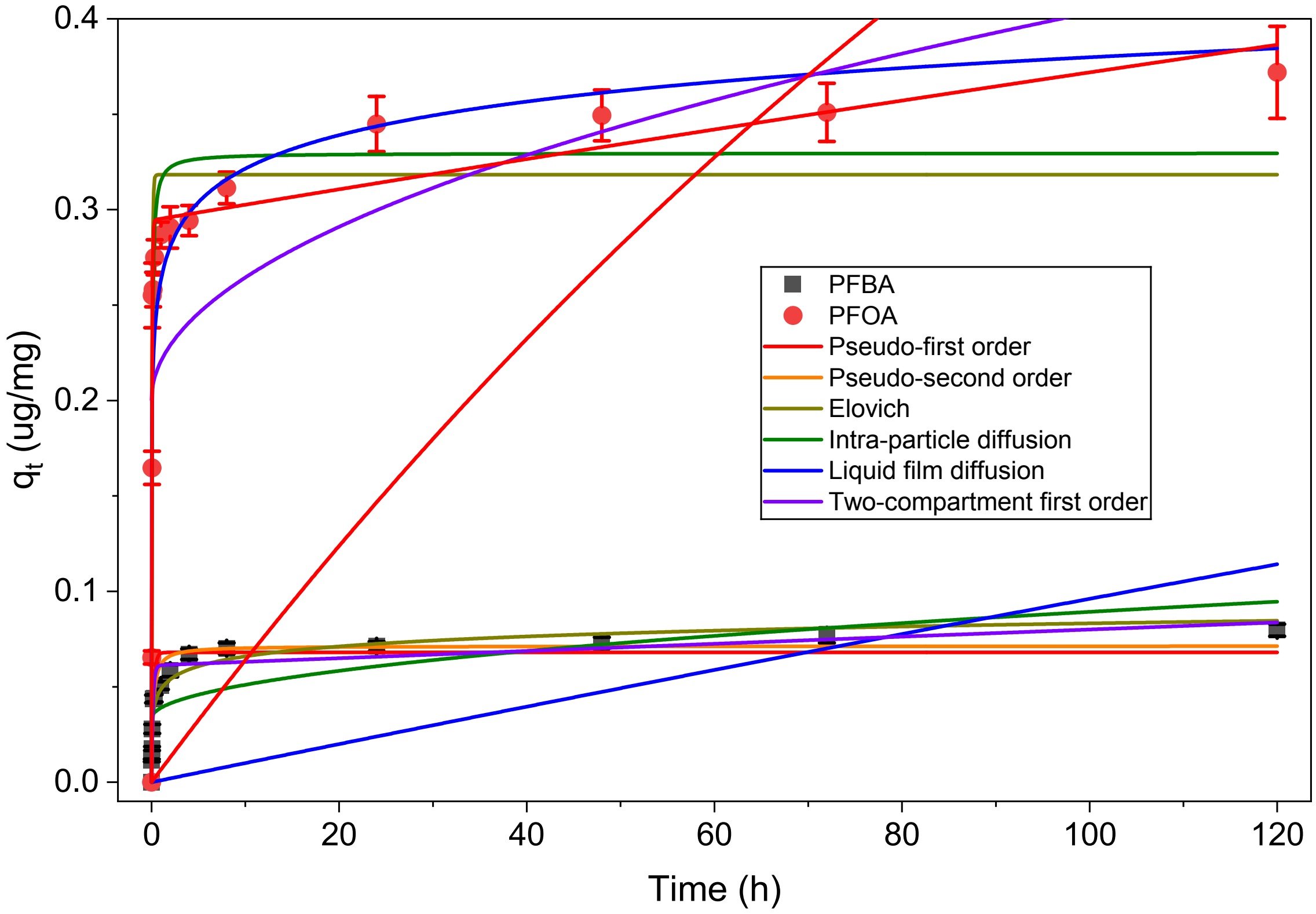

Figure S8. Experimental adsorption kinetics of PFOA and PFBA in the bisolute system on AF 900 at pH 3.0, together with the fitted curves of the pseudo-first-order, pseudo-second-order, Elovich, intraparticle diffusion, liquid film diffusion, and two-compartment first-order models.

Table S1. Adsorption isotherm, kinetic and thermodynamic models used in this study.

Models	Names and Equations
Kinetics	Pseudo-first order: $Q_t = Q_e\left(1 - exp(-K_1 t)\right)$ Pseudo-second order: $Q_t = \frac{K_2 Q_e^2 t}{1+K_2 Q_e t}$ Elovich: $Q_t = \left(\frac{1}{b}\right)lnab + \left(\frac{1}{b}\right)lnt,\ t_0 = \frac{1}{ab}$ Intra-particle diffusion: $Q_t = K_i\sqrt{t} + C_i$ Liquid film diffusion: $\ln(1-F) = -K_{fd}t, F = \frac{Q_t}{Q_e}$ Two-compartment first order: $\frac{Q_t}{Q_{t=\infty}} = F_{fast}\left(1 - exp^{-tK_{fast}}\right) + F_{slow}\left(1 - exp^{-tK_{slow}}\right),\ F_{fast} + F_{slow} = 1$
Isotherm	Freundlich isotherm: $Q_e = K_f C_e^{\frac{1}{nf}}$ Langmuir isotherm: $Q_e = \frac{Q_m K_L C_e}{1+K_L C_e}, R_L = \frac{1}{1+K_L C_0}$ Temkin isotherm: $Q_e = \frac{RT}{b_T}\ln\,(K_T C_e)$ Dubinin-Radushkevich: $Q_e = K_{DR}exp\left(-B_D\varepsilon_D^2\right)\ \ \varepsilon_D = RTln\left(1 + \frac{1}{C_e}\right); E = \frac{1}{\sqrt{2B_D}}$
Thermodynamics	$K_d = \frac{Q_e}{C_e}$ $\Delta G^o = -\mathrm{RT}\ln(K_d)$ $\ln(K_d) = -\frac{\Delta G^o}{RT} = \frac{\Delta S^o}{R} - \frac{\Delta H^o}{RT}$

[Note] where Q_t and Q_e represent the adsorption capacities (mg/g) at contact time t and at equilibrium, respectively; K_1 and K_2 are rate constants of pseudo-first-order and pseudo-second-order models, respectively; a denotes the chemisorption rate constant and *b* is the surface coverage constant; K_i is the intraparticle diffusion rate constant (mg $min^{0.5}$/g), and C_i is the intercept constant (mg/g); K_{fd} is the adsorption rate constant; F_{fast} and F_{slow} refer to the mass fraction of fast and slow compartments, respectively; K_{fast} and K_{slow} are the first-order transfer rate constants for the fast and slow compartments (h^{-1}); t is the contact time (h); C_e is the equilibrium concentration in liquid phase (mg/L), K_f and n_f are the Freundlich constants; Q_m is the maximum adsorption capacity (mg/g); K_L is the Langmuir constant (L/g); R_L is the separation factor; R is the universal gas constant (8.314 J/mol K); T is the absolute temperature (K); b_T is the Temkin constant; K_T is the equilibrium binding constant associated with the maximum binding energy; K_{DR} and B_D are the Dubinin-Radushkevich constants; ε_D is the Polanyi potential; E is the mean sorption energy; and ΔH^o, ΔS^o, and ΔG^o denote the standard enthalpy, entropy, and Gibbs free energy changes, respectively.

Table S2. Water quality of the ground water from Stephenville, TX, USA

Parameter	Concentration (ppm)
COD	99.1±5.5
TN	3.98±0.15
Nitrate	0.131±0.03
Ammonia	< LOD
Total phosphate	< LOD

Table S3. Surface elemental compositions of AF900 before and after PFOA and PFBA adsorption determined by XPS.

Sample	C (at.%)	O (at.%)	F (at.%)
AF900	80.77	19.23	ND
AF900 after PFOA adsorption	82.43	14.42	2.85
AF900 after PFBA adsorption	84.07	15.32	0.61

Table S4. Possible interactions between AF 900 and PFASs at various solution pH

pH	AF 900<br>(pH, $_{pzc}$ = 6.21)	PFOA, PFBA<br>(pK_a <1)	Possible interactions
1 < pH < 6.21	Positively charged, dominated	Negatively charged, dominated	Electrostatic attraction<br>π–π interaction<br>Hydrophobic interaction (pronounced for PFOA, limited for PFBA)<br>Pore-filling
pH > 6.21	Negatively charged, dominated	Negatively charged, dominated	Electrostatic repulsion<br>π–π interaction<br>Hydrophobic interaction (pronounced for PFOA, limited for PFBA)<br>Pore-filling

Table S5. Comparison of maximum adsorption capacities reported for PFOA and PFBA on pristine and activated/modified biochars.

PFAS	Category	Adsorbents	Specific surface area (m^2/g)	Biochar dosage	Initial concentration (mg/L)	Maximum Adsorption capacity (mg/g)	References
PFOA	Pristine	Alfalfa	242.29	0.25 g/L	0.025-2.0	6.369	This study
	Pristine	Sugarcane bagasse	127.07	0.67 g/L	0.5-4	1.59	[2]
	Pristine	Reed straw	731	0.2 g/L	0.1-250	17.9	[3]
	Pristine	Softwood	520	0.05 g/L	0.1-1.0	21.6	[4]
	Activated/ Modified	Granular Activated Carbon	726	0.05 g/L	0.1-1.0	35.7	[4]
	Activated/ Modified	Biosolid Carbon nanotube activated biochar	147.1	0.05 g/L	0.1-2.0	97.9	[5]
PFBA	Pristine	Alfalfa	242.29	0.25 g/L	0.025-2.0	0.436	This study
	Pristine	Reed straw	731	0.2 g/L	0.1-250	6.88	[3]
	Pristine	Reed straw	1133.9	0.5 g/L	0.05-10	15.63	[6]
	Activated/ Modified	$FeCl_3$ activated Reed straw	694.2	0.5 g/L	0.05-10	25.81	[6]
	Activated/ Modified	$FeCl_3$ activated Maize straw	79.5	1.25 g/L	0-100	10.1	[7]

[Note] * indicates the value calculated based on the maximum removal efficiency.

Table S6. Kinetic model parameters for the adsorption of PFOA and PFBA in the PFOA-PFBA binary system on AF 900 at pH 3.0

**Model**	**Parameter**	**PFOA**	**PFBA**
Pseudo-first-order	Q_e	0.31830	0.06802
	K_1	14.92616	5.01170
	R^2	0.92532	0.90470
Pseudo-second-order	Q_e	0.32960	0.07137
	K_2	65.81066	89.74568
	R^2	0.94516	0.95623
Elovich	Q_e^*	0.38446	0.08463
	a	817	4.90426
	b	39.45347	133.16659
	R^2	0.89128	0.95524
Intra-particle diffusion	K_i	0.02029	0.00559
	C_i	0.20031	0.03339
	R^2	0.43349	0.57462
Liquid film diffusion	K_{fd}	0.00661	0.00101
	R^2	-2.89421	-1.52633
Two-compartment first-order	F_{fast}	0.29444	0.06119
	K_{fast}	17.83362	6.45382
	K_{slow}	0.00116	2.02627E−4
	R^2	0.97818	0.95407

[Note] * is the Calculated value

References

(1) Jang, H. M.; Kan, E. A Novel Hay-Derived Biochar for Removal of Tetracyclines in Water. *Bioresour. Technol.* **2019**, *274*, 162–172. https://doi.org/10.1016/j.biortech.2018.11.081

(2) Pavithra, K.; Chakraborty, P. Adsorption of Perfluorooctanoic Acid from Aqueous Media Using an Engineered Sugarcane Bagasse Biochar–Chitosan Composite. *ChemEngineering* **2026**, *10* (2), 30. https://doi.org/10.3390/chemengineering10020030

(3) Liu, N.; Wu, C.; Lyu, G.; Li, M. Efficient Adsorptive Removal of Short-Chain Perfluoroalkyl Acids Using Reed Straw-Derived Biochar (RESCA). *Science of the Total Environment* **2021**, *798*. https://doi.org/10.1016/j.scitotenv.2021.149191

(4) Zhang, D.; He, Q.; Wang, M.; Zhang, W.; Liang, Y. Sorption of Perfluoroalkylated Substances (PFASs) onto Granular Activated Carbon and Biochar. *Environ. Technol.* **2021**, *42* (12), 1798–1809. https://doi.org/10.1080/09593330.2019.1680744

(5) Wu, Y.; Qi, L.; Chen, G. A Mechanical Investigation of Perfluorooctane Acid Adsorption by Engineered Biochar. *J. Clean. Prod.* **2022**, *340*. https://doi.org/10.1016/j.jclepro.2022.130742

(6) Liu, N.; Li, Y.; Zhang, M.; Che, N.; Song, X.; Liu, Y.; Li, C. Efficient Adsorption of Short-Chain Perfluoroalkyl Substances by Pristine and Fe/Cu-Loaded Reed Straw Biochars. *Science of the Total Environment* **2024**, *946*. https://doi.org/10.1016/j.scitotenv.2024.174223

(7) Liu, Z.; Zhang, P.; Wei, Z.; Xiao, F.; Liu, S.; Guo, H.; Qu, C.; Xiong, J.; Sun, H.; Tan, W. Porous Fe-Doped Graphitized Biochar: An Innovative Approach for Co-Removing per-/Polyfluoroalkyl Substances with Different Chain Lengths from Natural Waters and Wastewater. *Chemical Engineering Journal* **2023**, *476*. https://doi.org/10.1016/j.cej.2023.146888
